# Latent class analysis-derived classification improves the cancer-specific death stratification of molecular subtyping in colorectal cancer

**DOI:** 10.1038/s41698-023-00412-w

**Published:** 2023-06-23

**Authors:** Wen Zhou, Ming-Ming He, Feng Wang, Rui-Hua Xu, Fang Wang, Qi Zhao

**Affiliations:** 1 Department of Medical Oncology, Sun Yat-sen University Cancer Center, State Key Laboratory of Oncology in South China, Collaborative Innovation Center for Cancer Medicine, Sun Yat-sen University, 510060 Guangzhou, P. R. China; 2 Research Unit of Precision Diagnosis and Treatment for Gastrointestinal Cancer, Chinese Academy of Medical Sciences, 510060 Guangzhou, P. R. China; 3 Department of Molecular Diagnostics, Sun Yat-sen University Cancer Center, Sun Yat-sen University, 510060 Guangzhou, P. R. China

**Keywords:** Colorectal cancer, Tumour heterogeneity

## Abstract

The molecular subtypes of colorectal cancer (CRC) represent a comprehensive dissection of CRC heterogeneity. However, molecular feature-based classification systems have limitations in accurately prognosticating stratification due to the inability to distinguish cancer-specific deaths. This study aims to establish a classification system that bridges clinical characteristics, cause-specific deaths, and molecular features. We adopted latent class analysis (LCA) on 491,107 first primary CRC patients from the Surveillance, Epidemiology, and End Results (SEER) database to reveal hidden profiles of CRC. The LCA-derived classification scheme was further applied to The Cancer Genome Atlas (TCGA) to assess its effectiveness in improving the accurate stratification of molecular-based subtypes of CRC. Four classes were identified based on latent class analysis integrating demographic and clinicopathological information of CRC patients. The LCA-derived Class 1 (LCAC1) and the LCAC2 showed a high risk of dying from non-CRC, while patients in LCAC3 had a risk of dying from CRC 1.41 times that of LCAC1 (95% confidence interval [CI] = 1.39–1.43). LCAC4 had the lowest probability to die from non-CRC (hazard ratio [HR] = 0.22, 95% CI = 0.21–0.24) compared with LCAC1. Since the LCA-derived classification can identify patients susceptible to CRC-specific death, adjusting for this classification allows molecular-based subtypes to achieve more accurate survival stratification. We provided a classification system capable of distinguish CRC-specific death, which will improve the accuracy of consensus molecular subtypes for CRC patients’ survival stratification. Further studies are warranted to confirm the molecular features of LCA-derived classification to inform potential therapeutic strategies and treatment recommendations.

## Introduction

As one of the most common cancers worldwide^1,2^, colorectal cancer (CRC) is characterized by high heterogeneity concerning clinical and biological features, resulting in diverse treatment responses and prognoses^3,4^. Patients with CRC mainly exhibit three distinct phenotypes: microsatellite instability (MSI)^5^, chromosomal instability (CIN)^6^, and CpG island methylator phenotype (CIMP)^7^. These phenotypes interpreted the progression of colorectal carcinogenesis and demonstrated prognostic and predictive values^8–10^. Accurate classification of tumors is essential to inform treatment and predict prognosis^11^. In recent years, substantial efforts have been dedicated to CRC subtyping, but a more accurate classification is warranted to achieve an ideal stratification^12–14^.

The consensus molecular subtypes (CMS)^15^ of CRC represent the current best description of tumor heterogeneity at the gene-expression level^16^ and provide insight into predicting prognosis and treatment benefit^17–21^. For example, patients in CMS1 had the poorest overall survival (OS) and the combination of bevacizumab with Folinic acid, fluorouracil and oxaliplatin (FOLFOX) appeared more effective than cetuximab plus FOLFOX for both OS and progression-free survival (PFS)^22^. The first-line irinotecan (FIRE)-3 trial also showed that CMS was a strong independent prognostic factor for objective response rates (ORR), PFS, and OS^23^. Additionally, fluorouracil, folinic acid and irinotecan (FOLFIRI) plus bevacizumab was associated with inferior outcomes compared with FOLFIRI plus cetuximab for OS in CMS4 patients^23^. Recently, a study revealed two epithelial subtypes (intrinsic-consensus molecular subtype 2 [iCMS2] and iCMS3) based on single-cell transcriptomes and further proposed a refined “IMF” classification, which combines intrinsic epithelial subtype (I), microsatellite instability status (M), and fibrosis (F)^24^. The IMF classification represents the core epithelial intrinsic components of bulk CMS, refining the clinical stratification of CMS. Although these subtyping systems could effectively classify CRC patients with their expression patterns representing different molecular mechanisms of tumor genesis, they have an inadequate performance for patients’ risk stratification when competing risk events are present.

Competing risk events should be considered for accurate estimates of cancer survival in the frail population, who may die from other causes prior to the occurrence of cancer-caused death^25–27^. Non-cancer causes of death were high in patients with colorectal cancer^28^. The most common non-CRC causes of death (CODs) in CRC patients included heart disease, other types of malignancies, cerebrovascular disease, and chronic obstructive pulmonary disease (COPD)^29^. The probability of death from CRC will be overestimated since the competing CODs can lead to death before patients die from CRC^30^. Therefore, cause-specific survival is critical in guiding the treatment of CRC patients regarding future risk of death^31–33^. However, as COD information, which is essential for calculating cause-specific survival, is not always available, an alternative approach is needed to estimate survival in the presence of competing risks.

Latent class analysis (LCA)^34^ is a probabilistic modeling algorithm that allows clustering of data and statistical inference. LCA models work on the assumption that the observed distribution of the variables is the result of a finite latent (unobserved) mixture of underlying distributions^35^. To infer the latent groups, observed indicators were used in LCA models to identify the best patterns^36^. LCA-derived phenotyping has shown broad promise in identifying homogeneous subgroups within large heterogeneous populations recently^37,38^, such as acute respiratory distress syndrome (ARDS)^39,40^, asthma^41^, acute kidney injury^42^, and metastatic cancer^43^.

**Fig. 1:**
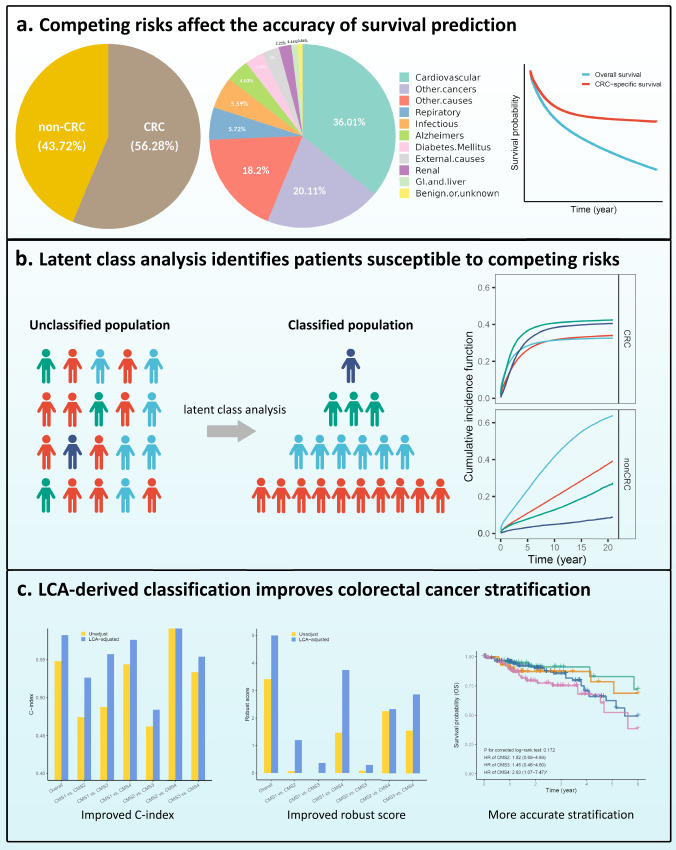
Overview of this study. **a** Competing risks affect the accuracy of survival prediction. **b** Latent class analysis identifies patients susceptible to competing risks. **c** LCA-derived classification improves colorectal cancer stratification.

To address the issue of inaccurate survival prediction due to the presence of competing risk events, we performed latent class analysis to refine CRC patients with factors that may affect cancer-specific survival (age, sex, race, tumor site, and stage)^44^ as indicator variables. The LCA-derived classification was subsequently assessed for cancer-specific survival stratification, enabling more accurate prognosis prediction in the absence of cause-of-death information (Fig. 1). In the current study, we proposed a four-part classification system for colorectal cancer using latent class analysis based on data from the Surveillance, Epidemiology and End Results (SEER) database. Additionally, we used the classification to distinguish CRC-specific death and to adjust the prediction of CRC prognosis by CMS. Molecular features were further investigated to provide evidence on treatment strategy and prognosis prediction.

## Results

### Patient population

A total of 491,107 first primary CRC patients were included (Supplementary Table 1, Supplementary Fig. 1). Throughout the entire follow-up period, a total of 268,034 patients died, with 43.72% of them attributed to non-CRC causes, especially cardiovascular disease. The range of follow-up period was 0–20.9 years, with a median follow-up of 3.9 years. Characteristics of most of the patients included were male (52.24%), age at diagnosis between 45–69 years (51.84%), non-Hispanic White (68.51%), married (56.71%), residing in metropolitan areas with a population greater than one million (57.16%), income between $50,000–$74,999 (48.19%), right-sided colon tumor (41.17%), stage III (28.40%), grade G2 (70.74%), and adenocarcinoma (72.53%).

### Characteristics of LCA-derived classification

**Fig. 2:**
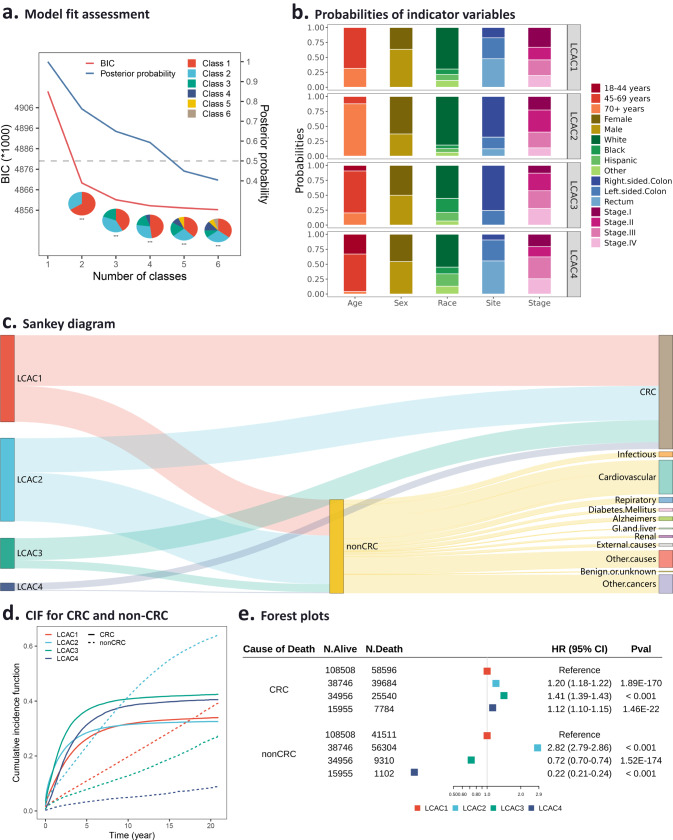
LCA-derived classification based on the SEER database. **a** Model fit assessment for latent class analysis. BIC, Bayesian information criterion. **b** Probabilities of indicator variables in each identified class. **c** Sankey diagram shows the proportion of causes of death in each class. **d** Cumulative incidence function (CIF) for colorectal cancer (CRC)-specific death and non-CRC death in each class. **e** Forest plots for CRC and non-CRC multi-state model. ***P < 0.001, **P < 0.01, *P < 0.05.

Patients’ hidden subgroups were identified using LCA model fit assessment (Fig. 2a, Supplementary Table 2). The best model fit selected was a four-class solution that had a low Bayesian information criterion (BIC) (4858200.0) and sample size-adjusted BIC (SABIC) (4858050.7), an entropy of 4.946, which indicating a clear separation of classes. The classes were named LCA-derived classes (LCACs), and their demographic and clinicopathological features were identified (Table 1).

**Table 1:** Latent class analysis of colorectal cancer patients in the SEER database (*N* = 491,107).

Characteristics	LCAC1 (47.63%) *N* = 208,615	LCAC2 (30.76%) *N* = 134,734	LCAC3 (15.94%) *N* = 69,806	LCAC4 (5.67%) *N* = 24,841
*Age at diagnosis*				
18–44 years	0	0	11.74	91.39
45–69 years	72.96	0	87.18	8.61
70+ years	27.04	100	1.09	0
*Sex*				
Female	31.51	65.12	53.84	52.57
Male	68.49	34.88	46.16	47.43
*Race/ethnicity*				
Non-Hispanic White	72.01	83.33	56.45	51.32
Non-Hispanic Black	7.43	5.12	24.95	10.32
Hispanic (All Races)	8.45	6.22	14.19	27.02
Other	12.11	5.32	4.41	11.34
*Tumor site*				
Right-sided colon	3.31	77.52	98.45	0.14
Left-sided colon	37.20	19.47	1.55	40.70
Rectum	59.49	3.01	0	59.16
*Stage*				
I	36.58	21.43	5.20	18.38
II	18.40	40.99	37.45	16.44
III	26.59	24.70	24.70	37.33
IV	18.43	12.88	32.65	27.85

Individuals that were not assigned to any class are not shown.

The contribution of indicator variables to latent classes was demonstrated in Fig. 2b. Specifically, LCAC1 (47.63% of patients) had a highest conditional probability of being 45–69 years old at diagnosis (72.96%), a high likelihood of being male (68.49%), and a tumor localized distally (left-sided colon and rectum, 96.69%). Patients in this group were less likely to die from CRC but more likely to die from non-CRC (Fig. 2c, d). Patients in LCAC2 (30.76% of patients) had the highest conditional probabilities of being diagnosed at 70+ years old (100%), female (65.12%), having a tumor localized in the colon (96.99%), and at staged I/II (62.42%). Patients in this group were more likely to die from non-CRC (Fig. 2c, d). Patients in LCAC3 (15.94% of patients) had the highest conditional probabilities of being diagnosed between 45–69 years old (87.18%), non-Hispanic Black (24.95%), and with a tumor localized proximally (right-sided colon, 98.45%). Patients in this group were more likely to die from CRC (Fig. 2c, d). Patients in LCAC4 (5.67% of patients) had the highest conditional probability of being diagnosed between 18–44 years old (91.39%), Hispanic (all races, 27.02%), having a tumor localized distally (left-sided colon and rectum, 99.86%), and at staged III/IV (65.18%). Most patients in this group died from CRC (Fig. 2c, d).

To confirm whether the LCA-derived classification was better than using the indicator variables alone, we analyzed the survival of patients aged 70+, as well as stage III/IV CRC patients and found significant differences in prognosis among different LCAC subgroups (Supplementary Fig. 2a, b). This indicated that the latent classes identified by the LCA can not only identify known effects but also recognize the potential interactions between indicator variables, resulting in more heterogeneity between classes and more homogeneity within classes. The features of each group remained consistent across subgroups, stratifying by age, tumor site, and tumor stage (Supplementary Fig. 2c). The trend in the proportion of causes of death suggested that as disease severity increases (from stage I to stage IV), the proportion of deaths from CRC increases and the proportion of deaths from non-CRC decreases (Supplementary Fig. 3).

The LCA-derived classification was associated with cause-specific survival. Compared with LCAC1, which had the lowest probability of dying from CRC, the risk of dying from CRC was increased in LCAC2 (hazard ratio [HR] = 1.20, 95% confidence interval [CI] = 1.18–1.22, *P* = 1.89 × 10^–170^), LCAC3 (HR = 1.41, 95% CI = 1.39–1.43, *P* < 0.001), and LCAC4 (HR = 1.12, 95% CI = 1.10–1.15, *P* = 1.46 × 10^–22^). Meanwhile, the risk of death from non-CRC was higher in LCAC2 (HR = 2.82, 95% CI = 2.79–2.86, *P* < 0.001), and lower in LCAC3 (HR = 0.72, 95% CI = 0.70–0.74, *P* = 1.52×10^–174^) and LCAC4 (HR = 0.22, 95% CI = 0.21–0.24, *P* < 0.001) compared with LCAC1 (Fig. 2e).

### Comparison of LCA-derived classification

**Fig. 3:**
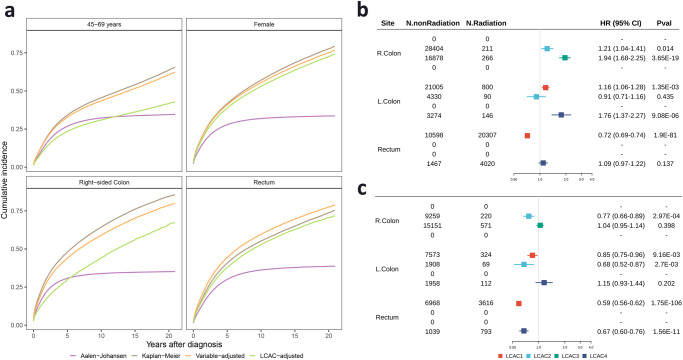
Application of LCA-derived classification. **a** Comparison of cumulative incidence estimators in patients aged 45–69 years, female, with right-sided colon cancer, and rectum cancer based on the SEER database. The color purple represents the Aalen-Johansen estimator, brown represents the Kaplan-Meier estimator, orange represents the adjustment of indicator variables (age at diagnosis, sex, race, tumor site, and stage), and green represents the adjustment of LCA-derived classification. Forest plot of radiation therapy in (**b**) stage III and (**c**) stage IV based on the SEER database. R.Colon, right-sided colon cancer; L.Colon, left-sided colon cancer; Rectum, rectum cancer.

In the presence of competing risk events, the Aalen-Johansen method accounts for the mutual exclusivity of competing events and the event of interest, providing an unbiased estimate of the cumulative incidence of the event of interest^45^. To assess whether the bias in survival prediction due to the presence of competing risk events (non-CRC death) can be reduced by adjusting the LCA-derived classification, we compared the cumulative incidence function (CIF) of death estimated by adjusting LCA-derived classification with the Aalen-Johansen estimator, the Kaplan-Meier estimator, and by adjusting indicator variables. We found that the estimator with adjusted LCA-derived classification provided a closer estimate to the Aalen-Johansen method in four subgroups, especially in the subgroup of individuals aged 45–69 years at diagnosis, suggesting that age was a stronger predictor of non-CRC death than other predictors (Fig. 3a).

### Clinical utility of LCA-derived classification

Notably, we observed worse survival for patients who received radiation therapy with resected, stage III right-sided colon cancer in the LCAC2 (HR = 1.21, 95% CI = 1.04–1.41, *P* = 0.014) and LCAC3 groups (HR = 1.94, 95% CI = 1.68–2.25, *P* = 3.65 × 10^–19^), as well as for left-sided colon cancer in the LCAC1 (HR = 1.16, 95% CI = 1.06–1.28, *P* = 1.35 × 10^–3^) and LCAC4 groups (HR = 1.76, 95% CI = 1.37–2.27, *P* = 9.08 × 10^–6^) (Fig. 3b). Conversely, better survival for patients who received radiation therapy in resected, advanced (stage IV) cases was observed for right-sided colon cancer in the LCAC2 group (HR = 0.77, 95% CI = 0.66–0.89, *P* = 2.97 × 10^–4^) and left-sided colon cancer in the LCAC1 (HR = 0.85, 95% CI = 0.75–0.96, *P* = 9.16 × 10^–3^) and LCAC2 groups (HR = 0.68, 95% CI = 0.52–0.87, *P* = 2.70 × 10^–3^) (Fig. 3c). These findings provided evidence that radiation therapy may not benefit colon cancer patients, particularly in non-advanced cases. The benefit of radiation therapy in patients with advanced colon cancer appeared to be limited to specific populations, such as right-sided colon cancer in the LCAC2 group (37.61% of stage IV right-sided colon cancer), and left-sided colon cancer in the LCAC1 and LCAC2 groups (66.12% and 16.55% of stage IV left-sided colon cancer, respectively). Similar results were also observed in patients with stage I and II (Supplementary Fig. 4).

### Validation of LCA-derived classification in the TCGA database

**Fig. 4:**
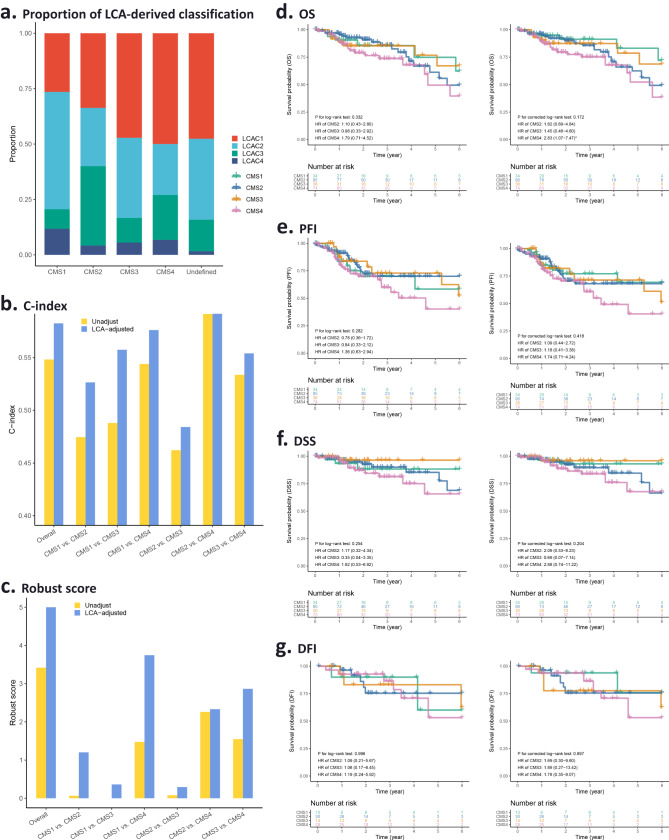
Adjustment performance of LCA-derived classification on consensus molecular subtypes (CMS) in the TCGA database. **a** Proportion of LCA-derived classification in each CMS subtype. **b** C-index for models distinguishing survival of CMS subtypes without and with adjustment of LCA-derived classification. **c** Robust score for comparison of survival between CMS subtypes without and with adjustment of LCA-derived classification. Cumulative survival probability (or survival function, survival rate) of CMS subtypes without (left panel) or with (right panel) adjustment of LCA-derived classification, for OS (**d**), PFI (**e**), DSS (**f**), and DFI (**g**), respectively.

To verify the consistency of the classification, we conducted latent class analysis in The Cancer Genome Atlas (TCGA) data. A total of 350 patients were included in the analysis, and a four-class model was found to be best fit the TCGA cohort (Supplementary Tables 3, 4, Supplementary Fig. 5). In TCGA, LCAC1 (LCAC1_TCGA_) corresponded to LCAC3 in SEER (LCAC3_SEER_, for the sake of brevity, in the rest of the article, those without subscripts refer to LCAC_SEER_), LCAC2_TCGA_ corresponded to LCAC2_SEER_, LCAC3_TCGA_ corresponded to LCAC4_SEER_, LCAC4_TCGA_ corresponded to LCAC1_SEER_. It is worth noting that 63 subjects who were unclassified by CMS were successfully assigned using the LCA-derived classification (Fig. 4a). After adjusting the LCA-derived classification, although we only observed statistically significant differences between survival curves of CMS1 and CMS4 in OS (HR = 2.83, 95% CI = 1.07–7.47, *P* = 0.036), we found improved performance of predicting the prognosis of CRC patients: the C-index and the robust score increased in the overall population and in each paired group (Fig. 4b–g). Since the LCA-derived classification can distinguish cause-specific survival, adjusting the LCA classification can improve the clinical applicability of CMS in prognosis prediction. Similar correction effects were observed in other classification systems as well (Supplementary Figs. 6, 7). The cumulative survival probabilities of different CRC molecular subtypes in each of the four LCA classes are shown in Supplementary Figs. 8–11.

To further refine the molecular characteristics of the LCA-derived classification, we compared mutation frequencies between LCAC subgroups (Supplementary Fig. 12a). LCAC2_TCGA_ (LCAC2_SEER_) and LCAC4_TCGA_ (LCAC1_SEER_) exhibited higher mutation burdens in *BRAF*, *CCDC168*, *USH2A*, and *KMT2D*. Additionally, LCAC4_TCGA_ (LCAC1_SEER_) showed higher mutation rates for *FAT3*, *SACS*, *TRPS1*, *PCDH15*, *VPS13B*, *DNAH10*, *GLI3*, *LRRK2*, and *RELN*. LCAC3_TCGA_ (LCAC4_SEER_) demonstrated a higher mutation burden in *APC*, *TP53*, and *FLG*, while a lower mutation rate for *PIK3CA*, *OBSCN*, and *KMT2B*. Furthermore, LCAC3_TCGA_ (LCAC4_SEER_) exhibited relatively lower overall mutation density than LCAC1_TCGA_ (LCAC3_SEER_, *P* < 0.01) and LCAC2_TCGA_ (LCAC2_SEER_, *P* < 0.0001), but higher clonal deletion score (CDS) and copy-neutral loss of heterozygosity (LOH) fraction than LCAC1_TCGA_ (LCAC3_SEER_, *P* < 0.001) and LCAC2_TCGA_ (LCAC2_SEER_, *P* < 0.01) (Supplementary Fig. 12b–d).

## Discussion

We developed a classifier using latent class analysis on SEER colorectal cancer patients. This LCA-derived classification integrated with demographic and clinicopathological information can identify subgroups in the CRC population. These subgroups consisted of individuals at high risk of death from CRC, thereby improving the clinical applicability of the consensus molecular subtype for CRC. The molecular characteristics of LCA-derived classification provided potential therapeutic targets/strategies and may help guide treatment and prognosis if validated in future studies.

Previous studies have highlighted the high probability of non-cancer-related mortality in patients with colorectal cancer^28,29^. Hence, it becomes crucial to provide appropriate treatment strategies for these patients, including measures to maintain general health^46–49^. Identifying individuals who are more likely to die from non-CRC among colorectal cancer patients can guide personalized treatment recommendations. Patients in the LCAC2 group had a high probability of being diagnosed at 70+ years old and were more likely to have chronic conditions, necessitating comprehensive supportive care for these patients. Conversely, patients in the LCAC3 and LCAC4 groups were more likely to be affected by CRC and may derive greater benefit from advancements in treatment.

In clinical practice, radiotherapy is generally considered beneficial for rectal cancer. However, in this study, we did not observe a significant benefit of radiotherapy in the LCAC4 group with stage III rectal cancer. This lack of benefit could be attributed to the higher proportion of T4b patients in the LCAC4 group (5.16% vs. 4.18% in LCAC1, *P* = 0.002). T4b CRC patients often present with symptoms such as partial obstruction and have a lower probability of achieving R0 resection with radiotherapy, which limits the potential survival benefit. Moreover, the LCAC4_SEER_ (corresponding to LCAC3_TCGA_) subgroup demonstrated a higher mutation burden in *TP53*, which has been associated with a decreased response to chemoradiation therapy^50^, indicating a potential reduced benefit from radiotherapy in this subgroup. Additionally, since early-onset colorectal cancer (EOCRC) patients have more aggressive tumor characteristics^51^, and the genetic background can influence the response to radiotherapy^52^, the different effects of radiotherapy among the LCAC groups may be attributed to the higher proportion of individuals in the LCAC3 and LCAC4 groups who are 18–44 years old at diagnosis (91.39% in LCAC4), non-Hispanic Black (24.95% in LCAC3), and Hispanic (27.02% in LCAC4).

The CMS classification of colorectal cancer holds promising potential for predicting prognosis and response to systemic therapy^17^. However, approximately 13% of patients with mixed features cannot be classified using CMS^15^. The LCA-derived classification based on demographic and clinicopathological information can serve as a supplement to the CMS system for classifying these unclassifiable patients to achieve accurate prognosis estimation and stratification. Furthermore, since the LCA-derived classification considered the cause of death, it can provide a correction to improve the prediction accuracy of CMS for CRC prognosis, thereby enhancing the clinical value of CMS in CRC.

Deciphering the molecular characteristics of cancers is crucial for understanding the underlying biological mechanisms and developing effective therapeutic strategies^53,54^. In our study, we observed that the LCAC4_TCGA_ subgroup had a higher proportion of young patients, and the proportion of LCAC4_TCGA_ was the highest in the MSI-H subgroup. This finding aligned with the association between early-onset colorectal cancers and Lynch syndrome^55^, which involves gene mutations in the mismatch repair pathway. Furthermore, patients in the LCAC4_TCGA_ subgroup exhibited significantly higher mutation rates in genes such as *FAT3*, *SACS*, *TRPS1*, *PCDH15*, *VPS13B*, *GLI3*, *LRRK2*, and *RELN*. Conversely, patients in the LCAC3_TCGA_ subgroup had lower mutation burdens in most genes but had the highest mutation burdens in *APC* and *TP53*. This suggested that mutant *APC* and *TP53* may serve as key driver genes in the carcinogenesis of this group, which exclusively consisted of patients with rectal cancer. Additionally, copy-neutral LOHs mutations have been reported to play a significant role in the early stages of tumor evolution^56^. Consistent with this, the LCAC4_TCGA_ subgroup had the lowest LOH fraction, as there was no stage I CRC patients included in this group. When comparing the features among the LCA-derived subgroups, we found that patients in the LCAC2_TCGA_ subgroup had a higher mutation density, indicating a high tumor mutation burden (TMB). These patients may potentially benefit from treatments targeting this feature. Recent studies have also identified somatic mutations as risk factors for the development of cardiovascular disease (CVD), with some mutations having a substantial impact on CVD development and severity^57^. Interestingly, patients in the LCAC3_TCGA_ subgroup had a higher clonal deletion score and loss of heterozygosity fraction, suggesting a higher likelihood of chromosomal instability in this subgroup.

This study presents a classification system that aims to address the impact of non-cancer-specific causes of death on prognostic prediction. However, there are several limitations that need to be acknowledged. Firstly, the lack of cause of death information in the TCGA database prevents direct validation of the adjustment effect of LCA-derived classification on cancer-specific cause of death. Secondly, although this classification system suggests potential therapeutic strategies, the underlying biological mechanisms and clinical value still require further confirmation. Thirdly, despite the large sample size, the generalizability of the results to other populations, such as Asians, may be limited as the SEER database primarily consists of data from White individuals. Fourthly, the prognosis of CRC is closely tied to treatment choice, particularly in the era of immunotherapy. However, both the SEER and TCGA database lack of comprehensive information on the results and severe adverse effects of immunotherapy for CRC. Further studies are warranted to validate the value of LCA-derived classification in the context of immunotherapy. Despite these limitations, the findings of this study provide a correction method for prognostic prediction in the presence of competing events when cause of death information is not available.

In conclusion, our analyses suggest that LCA-derived classification has the potential to aid in distinguishing cancer-specific death and improve the clinical utility of the consensus molecular subtype in colorectal cancer. The molecular characteristics identified through LCA-derived classification provide insights to potential therapeutic strategies and treatment recommendations. Prospective studies are warranted to validate the implementation of LCA-derived classification in clinical practice.

## Methods

### Ethical statements

The Sun Yat-sen University Cancer Center (SYSUCC) Institutional Review Board (IRB) waived the requirement for a Research Data Agreement and informed consent, in accordance with the principles of the Declaration of Helsinki.

### Patient population

We conducted a population-based retrospective study using data from the SEER Program, which collects and publishes cancer incidence and survival data from population-based cancer registries covering approximately 48% of the U.S. population. The collected data includes patient demographics, primary tumor site, tumor morphology and stage at diagnosis, the first course of treatment, and follow-up for vital status^58^.

We extracted data on all first primary colorectal cancers diagnosed between 2000 and 2020 from the SEER Research Data 17^59^ using SEER*Stat 8.4.1 (RRID: SCR_003293)^60^. Diagnosis was based on coding in the International Classification of Diseases for Oncology 3rd edition [ICD-O-3] codes, 8000–8982. Subjects were excluded if their diagnoses were not confirmed by positive histology. We also excluded subjects diagnosed before 18 years old, with staged carcinoma in situ, with tumors located in the appendix, or those with unknown age at diagnosis, race/ethnicity, stage, tumor sites, cause of death, and/or date of death (Supplementary Fig. 1).

### Definitions

Available demographic characteristics included age at diagnosis, sex, race, marital status, residential area (rural or urban), and household income. Clinicopathological information for colorectal cancer included the year of diagnosis, American Joint Committee on Cancer (AJCC) TNM stage, tumor site, grade, histology, vital status at last follow-up, and cause of death. The TNM stage was based on AJCC 3rd stage codes for patients diagnosed between 2000 and 2003, AJCC 6th stage codes for patients diagnosed between 2004 and 2009, AJCC 7th stage codes for patients diagnosed between 2010 and 2015, SEER combined stage for patients diagnosed in 2016 and 2017, and AJCC 8th stage codes for patients diagnosed in 2018 and 2020^61^. Right-sided colon cancer was defined based on tumor site as those occurring from the cecum up to but not including the splenic flexure. Left-sided colon cancer was defined as those occurring from the splenic flexure to the sigmoid colon.

Follow-up from diagnosis was defined as the interval between cancer diagnosis and death from any cause, the last follow-up, or the end of the study on December 31, 2022, whichever came first. SEER Cause-specific Death Classification was classified into two groups: death from CRC and death from non-CRC. CODs were defined by the SEER Cause of Death Recode variable from death certificates^62^. Non-cancer causes were categorized into 26 groups, and we further consolidated them into nine categories: infection, CVD, respiratory disease, gastrointestinal and liver disease, renal disease, diabetes mellitus (DM), Alzheimer’s, external causes, and other causes. Besides, non-CRC causes also included other cancers and deaths from in situ, benign or unknown behavior neoplasms.

### Latent class analysis

The LCA is one of the finite mixture modeling techniques that allow investigators to determine if unobserved groups exist within a population. LCA models work on the assumption that there are underlying unobserved variables that divide a population into mutually exclusive and collectively exhaustive latent classes^63^. These models identify solutions that best describe these latent classes by utilizing a set of observed indicators and estimate the parameters by maximizing likelihood or employing the Bayesian method. In simple terms, LCA is a probabilistic method of unsupervised clustering. Once the model has been fitted, the probability of class membership is estimated for each observation in the cohort, and these probabilities can be used to assign a class.

We performed LCA on the SEER data to identify hidden subgroups in colorectal cancer. The observed indicators used in LCA included patient demographic characteristics and clinicopathological information such as age at diagnosis, sex, race, tumor site, and stage. Age at diagnosis was categorized into three groups: 18–44 years, 45–69 years, and 70+ years. Sex was assessed as a dichotomous variable (male and female), and race was classified as non-Hispanic White, non-Hispanic Black, Hispanic (All Races), and other. Tumor site was categorized as right-sided colon, left-sided colon, and rectum. Stage was categorized as I, II, III, or IV.

We created multiple models based on the number of classes (i.e., 1-, 2-, 3-, 4-, 5-, 6-class solutions), compared their model fit, and selected the model that met the following criteria: (i) lower values of BIC^64^ and SABIC^65^; if the sample size was less than 500, the Akaike information criteria (AIC)^66^ was used instead. (ii) Entropy not less than 0.8, which indicates an acceptable quality of classification and a good indication for class separation^36^. (iii) A statistically significant test of the probability that a model with *k* classes fits better than a model with *k*-1 classes using the Lo-Mendel-Rubin likelihood ratio test (LMR)^67^. (iv) Average posterior probabilities of subgroup membership greater than or equal to 0.5 for each subgroup^36^. (v) The smallest class has more than 5% of the individuals in the entire population^68^. For each participant, a posterior probability, which predicts the likelihood of belonging to each of the identified classes, was estimated. A probability cutoff of greater than or equal to 0.5 was used to assign a class to each participant. The class with the largest posterior probability was assigned to that participant. All LCAs were conducted using poLCA in R version 4.0.3 (RRID: SCR_003005). The poLCAParallel^69^, a reimplementation of poLCA, was used to speed up the running. The latent class models were estimated with the default parameters (graphs=FALSE, tol=1e-10, na.rm=TRUE, calc.se=TRUE), except for nclass=1–6, maxiter=1000, and nrep=30. Additionally, as the numerical order of the estimated latent classes in the model output is determined solely by the start values of the expectation-maximization (EM) algorithm, the poLCA.reorder command was used to ensure consistency in the category labels assigned to each latent class in each run.

### Application and validation of LCA-derived classification

In the presence of competing events, the CIF was preferred to calculate. The Aalen-Johansen method, which is based on a multi-state model and provides unbiased estimates of CIF, was considered the gold standard for estimating CIF in the presence of censored competing events^70,71^. In addition, we compared the correction effect of LCA-derived classification on CIF with CIF estimated by Kaplan-Meier and CIF adjusted by indicator variables (age at diagnosis, sex, race, tumor site, and stage).

To further assess the LCA-derived classification, we calculated the probabilities of class assignment for individuals in the TCGA (RRID: SCR_003193) using the LCA model with the optimal number of classes. Patients were assigned to a class based on their highest probability. We then compared the survival of the four CMS subtypes with adjustment of LCA-derived classification to explore whether it could improve the clinical utility of CMS in colorectal cancer by considering non-CRC causes of death.

### Assessment of molecular characteristics

To investigate the molecular characteristics of the LCA-derived classes, we compared the mutation profiles between classes for patients in TCGA. Somatic mutation calling data was obtained using the GDC Data Transfer Tool and the UCSC Xena platform (RRID: SCR_018938)^72^. Several quality-control filters were applied to the mutations: (i) sequencing depth ≥ 20; (ii) sequence reads in support of the variant call ≥ 5; (iii) variant allele frequency (VAF) ≥ 0.02; and (iv) identified in at least two of the four callers (MuSE, MuTect2, SomaticSniper [RRID: SCR_005108], and VarScan2 [RRID: SCR_006849]). Other molecular features such as microsatellite instability, somatic copy number alterations, tumor ploidy, CDS, mutational signatures, and stemness index were obtained from previous studies^73^.

### Statistical analysis

Characteristics of subjects were compared among subgroups of colorectal cancer using χ^2^ tests or Fisher’s exact tests where appropriate. Survival analysis was performed using Kaplan-Meier, and survival curves were compared using the log-rank test. The CIF was estimated for CRC-related deaths and non-CRC-related deaths. The multi-state model was used to assess cancer-specific survival (CSS) in CRC^70^. To improve the prognostic stratification of molecular subtypes, including CMS, the LCA-derived classification was used to adjust the survival curves. Details of the approaches for adjusting survival curves have been described elsewhere^74–76^. Briefly, two main approaches were used for adjusting survival curves: marginal analysis and conditional approach. The marginal analysis involves reweighting the database to obtain balanced subgroups and then analyzing survival using the reformulated data. On the contrary, the conditional approach predicts the curves first and then averages the predictions for each subgroup. In this study, the survival curves were adjusted using rescaled weights^76^, which belongs to the marginal method. The robust score test from *coxph*, corresponds to a log-rank test corrected for weighting, was used to compare the adjusted survival curves. In addition, to visualize the correction effect of the LCA-derived classification on the survival curve, we compared the C-index and robust score before and after adjustment. All statistical analyses were performed in R version 4.0.3. A *P*-value less than 0.05 was considered statistically significant.

### Reporting summary

Further information on research design is available in the Nature Research Reporting Summary linked to this article.

## Supplementary information

Supplementary information

REPORTING SUMMARY

## Data Availability

The data analyzed in this study were obtained from SEER Program SEER*Stat Database (Incidence - SEER Research Data, 17 Registries, Nov 2022 Sub [2000–2020] - Linked To County Attributes - Time Dependent [1990–2021] Income/Rurality, 1969–2021 Counties, National Cancer Institute, DCCPS, Surveillance Research Program, released April 2023, based on the November 2022 submission), of which detailed information is available on https://seer.cancer.gov/data-software/documentation/seerstat/nov2022/. A SEER*Stat account is needed to access the SEER Research Data (for personal use), with acknowledge of the SEER Research Data Use Agreement, SEER Treatment Data Limitations, and Best Practices Assurance. The demographic and clinicopathological information of the TCGA cohort were obtained from the Genomic Data Commons (GDC) program which provided by a previous study^73^ (https://gdc.cancer.gov/about-data/publications/Pan-GI) and the UCSC Xena platform (Cohort names: “GDC TCGA Colon Cancer” and “GDC TCGA Rectal Cancer” at https://xenabrowser.net/datapages/). The survival information was downloaded using the UCSC Xena platform (Cohort name: “TCGA Colon and Rectal Cancer” at https://xenabrowser.net/datapages/). The somatic mutation calling of the TCGA cohort was downloaded using the GDC Data Transfer Tool (UUID for colon cancer: 70cb1255-ec99-4c08-b482-415f8375be3f, 03652df4-6090-4f5a-a2ff-ee28a37f9301, 70835251-ddd5-4c0d-968e-1791bf6379f6, and 8177ce4f-02d8-4d75-a0d6-1c5450ee08b0; UUID for rectal cancer: ec8ec3ad-f08d-46eb-9571-42806e304b37, faa5f62a-2731-4867-a264-0e85b7074e87, e48ffb82-9208-4be3-8a47-0a1168a07054, and b2689e8f-3b64-4214-8a87-dc7e7cf6fe5e).

## Code availability

All code for data cleaning and analysis associated with the current submission is available at https://github.com/wenzhou-cn/LCAC.
