# Publisher Correction: Latent class analysis-derived classification improves the cancer-specific death stratification of molecular subtyping in colorectal cancer

**DOI:** 10.1038/s41698-023-00416-6

**Published:** 2023-07-05

**Authors:** Wen Zhou, Ming-Ming He, Feng Wang, Rui-Hua Xu, Fang Wang, Qi Zhao

**Affiliations:** 1 Department of Medical Oncology, Sun Yat-sen University Cancer Center, State Key Laboratory of Oncology in South China, Collaborative Innovation Center for Cancer Medicine, Sun Yat-sen University, 510060 Guangzhou, P. R. China; 2 Research Unit of Precision Diagnosis and Treatment for Gastrointestinal Cancer, Chinese Academy of Medical Sciences, 510060 Guangzhou, P. R. China; 3 Department of Molecular Diagnostics, Sun Yat-sen University Cancer Center, Sun Yat-sen University, 510060 Guangzhou, P. R. China

**Keywords:** Colorectal cancer, Tumour heterogeneity

Correction to: *npj Precision Oncology* 10.1038/s41698-023-00412-w, published online 23 June 2023

In this article the author affiliation links for authors’ Ming-Ming He, Feng Wang, Rui-Hua Xu, Qi Zhao were incorrectly given as ‘3’ but should have been ‘2’ and Fang Wang the affiliation link 2 should have been 3. The original article has been corrected.

