# Supplementary material for: Latent class analysis-derived classification improves the cancer-specific death stratification of molecular subtyping in colorectal cancer
Source: NPJ Precis Oncol. 2023 Jun 23;7:60. doi: 10.1038/s41698-023-00412-w (PMC10290127; doi:10.1038/s41698-023-00412-w)
Supplement: Supplementary file 1 — Supplementary information [file 41698_2023_412_MOESM1_ESM.pdf]

Latent class analysis-derived classification improves the cancer-specific death stratification of molecular subtyping in colorectal cancer

Wen Zhou[#], Ming-Ming He[#], Feng Wang, Rui-Hua Xu, Fang Wang*, Qi Zhao*

Contents

Supplementary Table 1. Demographic and clinicopathological characteristics of CRC patients in the SEER database

Supplementary Table 2. Latent class analysis model fit assessment in the SEER database

Supplementary Table 3. Latent class analysis model fit assessment in the TCGA database

Supplementary Table 4. Latent class analysis of colorectal cancer patients in the TCGA database

Supplementary Figure 1. Inclusion and exclusion criteria of patients in the SEER database.

Supplementary Figure 2. Survival probabilities and cumulative incidence in subgroups.

Supplementary Figure 3. Trend of the proportion of cause of deaths in each class for stage I to stage IV based on the SEER database.

Supplementary Figure 4. Forest plot of radiation therapy in stage I and stage II based on the SEER database.

Supplementary Figure 5. LCA-derived classification based on the TCGA database.

Supplementary Figure 6. Adjustment performance of LCA-derived classification on microsatellite instability (MSI) in the TCGA database.

Supplementary Figure 7. Adjustment performance of LCA-derived classification on molecular subtypes in the TCGA database.

Supplementary Figure 8. Cumulative survival probabilities (OS) of different CRC molecular subtypes in each of the four LCA classes in the TCGA database.

Supplementary Figure 9. Cumulative survival probabilities (PFI) of different CRC molecular subtypes in each of the four LCA classes in the TCGA database.

Supplementary Figure 10. Cumulative survival probabilities (DSS) of different CRC molecular subtypes in each of the four LCA classes in the TCGA database.

Supplementary Figure 11. Cumulative survival probabilities (DFI) of different CRC molecular subtypes in each of the four LCA classes in the TCGA database.

Supplementary Figure 12. Molecular features of LCA-derived classification based on the TCGA dataset.

Supplementary Table 1. Demographic and clinicopathological characteristics of CRC patients in the SEER database

Characteristics	No. of patients (%)	No. of deaths (%)	Deaths from CRC (%)	Deaths from non-CRC (%)
Total	491,107	268,034	150,840	117,194
Age at diagnosis				
18-44 years	30893 (6.29)	10967 (4.09)	9535 (6.32)	1432 (1.22)
45-69 years	254581 (51.84)	108631 (40.53)	73811 (48.93)	34820 (29.71)
70+ years	205633 (41.87)	148436 (55.38)	67494 (44.75)	80942 (69.07)
Sex				
Female	234537 (47.76)	126981 (47.37)	70585 (46.79)	56396 (48.12)
Male	256570 (52.24)	141053 (52.63)	80255 (53.21)	60798 (51.88)
Race				
non-Hispanic White	336447 (68.51)	191874 (71.59)	102257 (67.79)	89617 (76.47)
non-Hispanic Black	53704 (10.94)	30538 (11.39)	19174 (12.71)	11364 (9.7)
Hispanic (All Races)	55240 (11.25)	25201 (9.4)	16450 (10.91)	8751 (7.47)
Other	45716 (9.31)	20421 (7.62)	12959 (8.59)	7462 (6.37)
Martital				
Married (including common law)	265759 (56.71)	132260 (51.53)	75562 (52.23)	56698 (50.63)
Unmarried or Domestic Partner	995 (0.21)	275 (0.11)	209 (0.14)	66 (0.06)
Separated	4943 (1.05)	2590 (1.01)	1618 (1.12)	972 (0.87)
Single (never married)	74370 (15.87)	38899 (15.16)	25378 (17.54)	13521 (12.07)
Divorced	44203 (9.43)	24351 (9.49)	14670 (10.14)	9681 (8.64)
Widowed	78347 (16.72)	58271 (22.7)	27225 (18.82)	31046 (27.72)
Residential area				
Metropolitan areas greater than 1M population	280702 (57.16)	149988 (55.96)	85891 (56.94)	64097 (54.7)
Metropolitan areas of 250K to 1M population	100875 (20.54)	54433 (20.31)	30249 (20.05)	24184 (20.64)
Metropolitan areas of less than 250K population	40900 (8.33)	23492 (8.76)	12852 (8.52)	10640 (9.08)
Adjacent to a metropolitan area	39645 (8.07)	23328 (8.7)	12724 (8.44)	10604 (9.05)
Nonmetropolitan	27896 (5.68)	16233 (6.06)	8805 (5.84)	7428 (6.34)
Unknown/missing/no match	1061 (0.22)	547 (0.2)	313 (0.21)	234 (0.2)

Supplementary Table 1. Demographic and clinicopathological characteristics of CRC patients in the SEER database (continued)

Characteristics	No. of patients (%)	No. of deaths (%)	Deaths from CRC (%)	Deaths from non-CRC (%)
Household income				
< $35,000	5328 (1.08)	2994 (1.12)	1751 (1.16)	1243 (1.06)
$35,000 - $49,999	49134 (10.01)	27949 (10.43)	15913 (10.55)	12036 (10.27)
$50,000 - $74,999	236667 (48.19)	133798 (49.92)	75930 (50.34)	57868 (49.38)
$75,000+	199950 (40.72)	103280 (38.53)	57240 (37.95)	46040 (39.29)
Tumor site				
Right-sided Colon	202195 (41.17)	119030 (44.41)	61763 (40.95)	57267 (48.87)
Left-sided Colon	141086 (28.73)	73843 (27.55)	41797 (27.71)	32046 (27.34)
Rectum	147826 (30.1)	75161 (28.04)	47280 (31.34)	27881 (23.79)
T				
T1	85377 (17.39)	33049 (12.33)	10971 (7.27)	22078 (18.84)
T2	64171 (13.07)	27846 (10.39)	7659 (5.08)	20187 (17.23)
T3	232929 (47.44)	124590 (46.49)	65554 (43.47)	59036 (50.38)
T4	68974 (14.05)	46879 (17.49)	35496 (23.54)	11383 (9.71)
TX	39580 (8.06)	35630 (13.3)	31132 (20.64)	4498 (3.84)
N				
N0	278574 (56.73)	133914 (49.97)	52019 (34.49)	81895 (69.89)
N1	109927 (22.39)	57964 (21.63)	38735 (25.68)	19229 (16.41)
N2	61407 (12.51)	41569 (15.51)	33698 (22.34)	7871 (6.72)
N3	4359 (0.89)	3679 (1.37)	2413 (1.6)	1266 (1.08)
NX	36764 (7.49)	30868 (11.52)	23947 (15.88)	6921 (5.91)
M				
M0	392061 (79.84)	183845 (68.6)	76881 (50.98)	106964 (91.28)
M1	98970 (20.16)	84149 (31.4)	73931 (49.02)	10218 (8.72)

Supplementary Table 1. Demographic and clinicopathological characteristics of CRC patients in the SEER database (continued)

Characteristics	No. of patients (%)	No. of deaths (%)	Deaths from CRC (%)	Deaths from non-CRC (%)
Stage				
I	122119 (24.87)	47009 (17.54)	9932 (6.58)	37077 (31.64)
II	130494 (26.57)	64998 (24.25)	23335 (15.47)	41663 (35.55)
III	139488 (28.4)	71852 (26.81)	43623 (28.92)	28229 (24.09)
IV	99006 (20.16)	84175 (31.4)	73950 (49.03)	10225 (8.72)
Grade				
G1	42876 (9.86)	18801 (7.88)	7477 (5.74)	11324 (10.46)
G2	307769 (70.74)	163176 (68.42)	85848 (65.92)	77328 (71.42)
G3	76020 (17.47)	50977 (21.37)	33187 (25.48)	17790 (16.43)
G4	8395 (1.93)	5552 (2.33)	3720 (2.86)	1832 (1.69)
Histology				
Adenocarcinoma	356215 (72.53)	198076 (73.9)	117269 (77.74)	80807 (68.95)
Papillary	44024 (8.96)	21353 (7.97)	8122 (5.38)	13231 (11.29)
Adenomatous Polyp	30075 (6.12)	12473 (4.65)	4153 (2.75)	8320 (7.1)
Mucinous	38231 (7.78)	24446 (9.12)	13645 (9.05)	10801 (9.22)
Carcinoid	8678 (1.77)	2010 (0.75)	975 (0.65)	1035 (0.88)
Signet Ring	4820 (0.98)	3774 (1.41)	2851 (1.89)	923 (0.79)
Squamous	1943 (0.4)	959 (0.36)	430 (0.29)	529 (0.45)
Medullary	692 (0.14)	300 (0.11)	147 (0.1)	153 (0.13)
Small Cell	419 (0.09)	361 (0.13)	244 (0.16)	117 (0.1)
Other	6010 (1.22)	4282 (1.6)	3004 (1.99)	1278 (1.09)

Supplementary Table 2. Latent class analysis model fit assessment in the SEER database

Class	BIC	SABIC	Entropy	P_{LMR}	$P_{average}$	$Min_{proportion}$
1-Class Solution	4913985.2	4913950.2	5.003	-	1.00	100.00
2-Class Solution	4869324.2	4869251.1	4.958	< 0.001	0.762	35.53
3-Class Solution	4861119.3	4861008.0	4.950	< 0.001	0.649	25.23
**4-Class Solution**	**4858200.0**	**4858050.7**	**4.946**	**< 0.001**	**0.593**	**14.09**
5-Class Solution	4857116.8	4856929.3	4.945	1.40E-251	0.449	10.69
6-Class Solution	4856275.8	4856050.2	4.944	9.38E-201	0.404	8.22

$P_{average}$: the smallest average posterior probabilities of subgroup membership; $Min_{proportion}$: the proportion of whole population the smallest class has.

Supplementary Table 3. Latent class analysis model fit assessment in the TCGA database

Class	AIC	BIC	SABIC	Entropy	P_{LMR}	$P_{average}$	$Min_{proportion}$
1-Class Solution	3231.7	3270.2	3238.5	4.588	-	1.00	100.00
2-Class Solution	3220.5	3301.5	3234.9	4.540	9.50E-04	0.873	37.00
3-Class Solution	3217.5	3341.0	3239.5	4.507	0.015	0.869	26.71
**4-Class Solution**	**3216.2**	**3382.1**	**3245.7**	**4.471**	**0.024**	**0.652**	**8.63**
5-Class Solution	3223.0	3431.3	3260.0	4.449	0.210	0.736	10.50
6-Class Solution	3228.6	3479.3	3273.1	4.424	0.161	0.624	7.21

$P_{average}$: the smallest average posterior probabilities of subgroup membership; $Min_{proportion}$: the proportion of whole population the smallest class has.

Table S4. Latent class analysis of colorectal cancer patients in the TCGA database (N = 350*)

Characteristics	$LCAC1_{TCGA}$ (40.79%) N=135	$LCAC2_{TCGA}$ (31.12%) N=103	$LCAC3_{TCGA}$ (23.26%) N=77	$LCAC4_{TCGA}$ (4.83%) N=16
Age at diagnosis				
18-44 years	11.11	0	14.29	25.00
45-69 years	88.89	0	51.95	62.50
70+ years	0	100	33.77	12.50
Sex				
Female	60.74	44.66	46.75	18.75
Male	39.26	55.34	53.25	81.25
Race/Ethnicity				
non-Hispanic White	64.44	84.47	100	18.75
non-Hispanic Black	35.56	15.53	0	0
Other	0	0	0	81.25
Tumor site				
Right-sided Colon	54.81	70.87	0	68.75
Left-sided Colon	42.22	29.13	0	25.00
Rectum	2.96	0	100	6.25
Stage				
I	19.26	15.53	11.69	0
II	17.78	47.57	27.27	87.50
III	42.96	25.24	42.86	12.50
IV	20.00	11.65	18.18	0

*Individuals that were not assigned to any class were not shown.

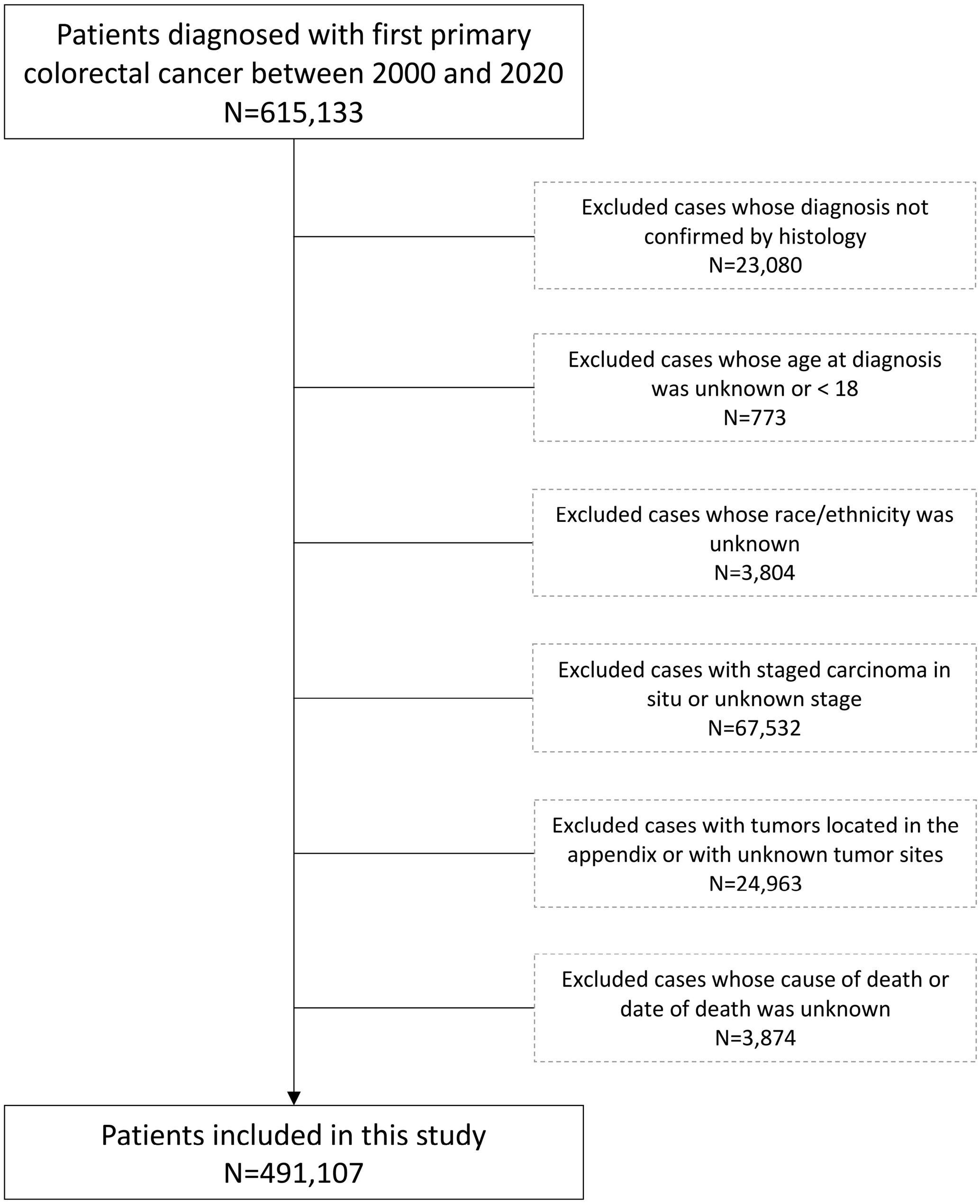

Supplementary Figure 1. Inclusion and exclusion criteria of patients in the SEER database.

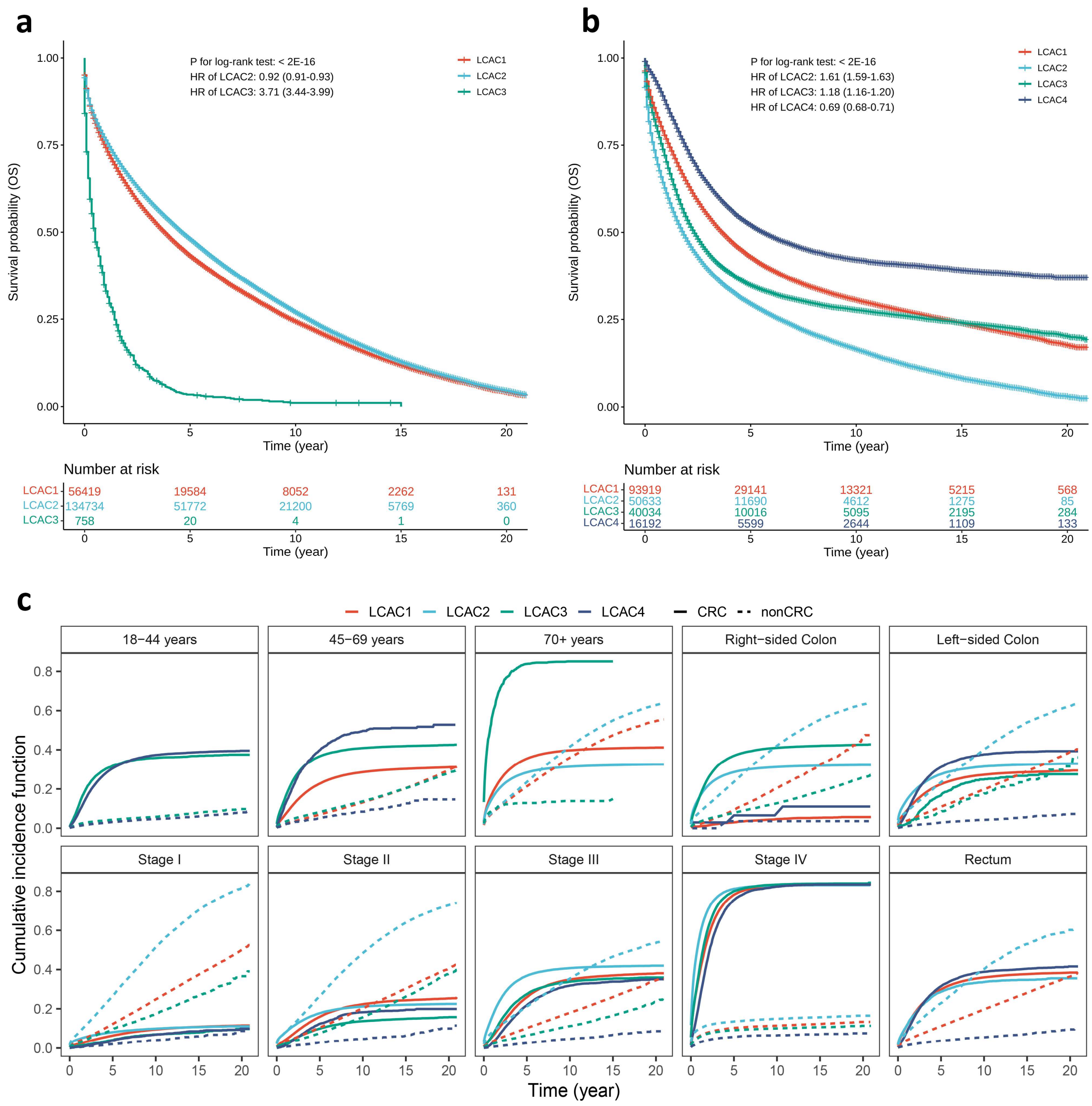

Supplementary Figure 2. Survival probabilities and cumulative incidence in subgroups. (a) Survival probability for 70+ years old colorectal cancer (CRC) patients in the SEER database. (b) Survival probability for stage III/IV CRC patients. (c) Cumulative incidence function (CIF) for CRC-specific death and non-CRC death in each class stratified by age (18-44 years, 45-69 years, and 70+ years), tumor site (right-sided colon, left-sided colon, and rectum), and stage (I/II/III/IV).

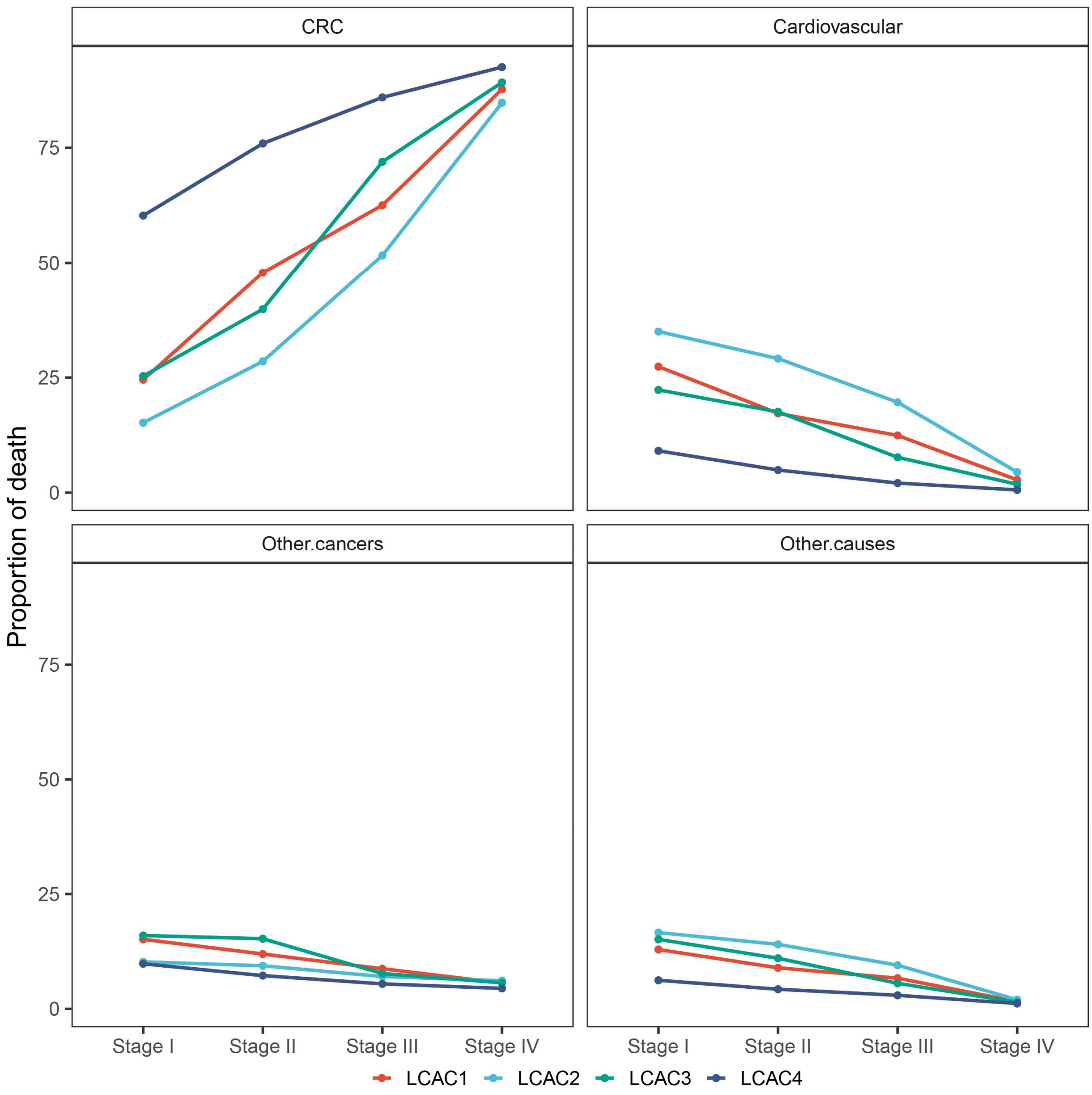

Supplementary Figure 3. Trend of the proportion of cause of deaths in each class for stage I to stage IV based on the SEER database.

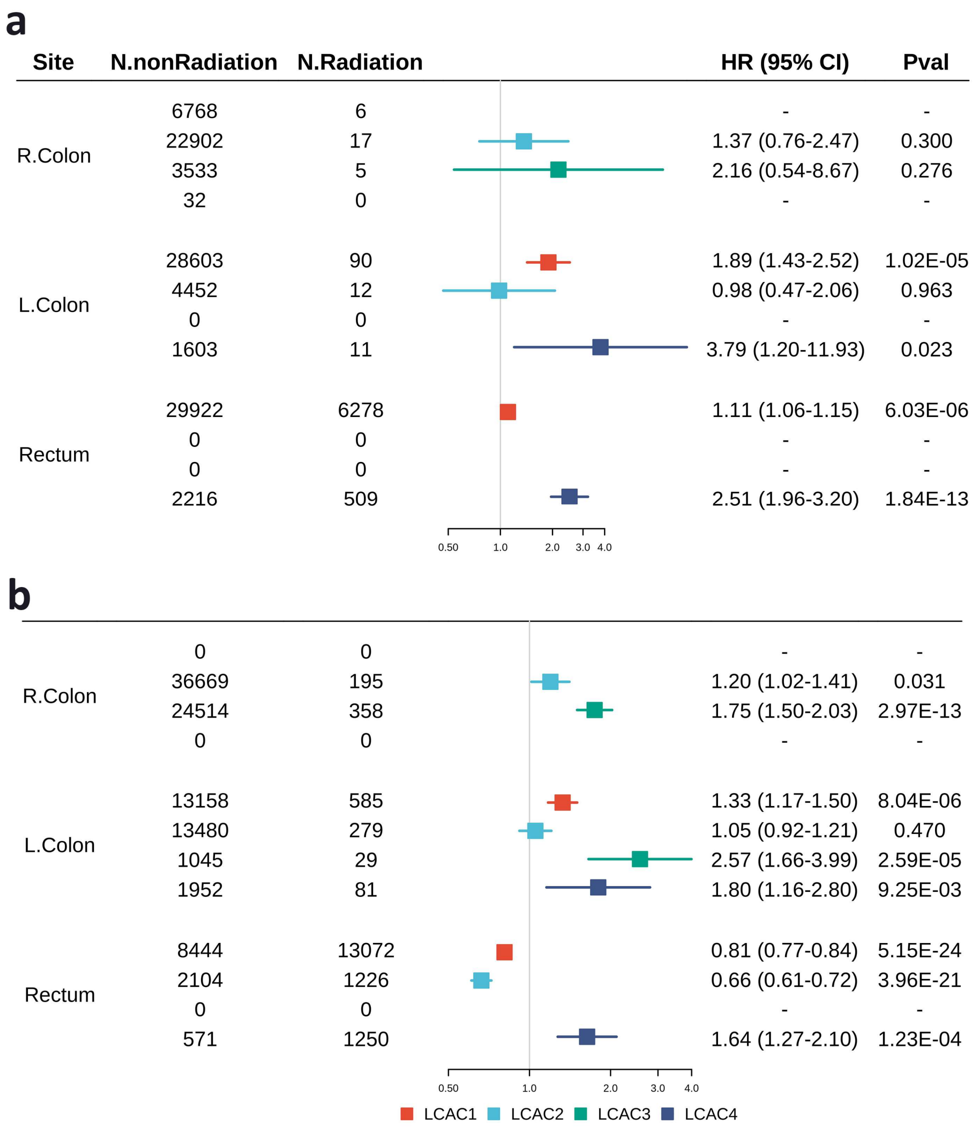

Supplementary Figure 4. Forest plot of radiation therapy in (a) stage I and (b) stage II based on the SEER database. R.Colon, right-sided colon cancer; L.Colon, left-sided colon cancer; Rectum, rectum cancer.

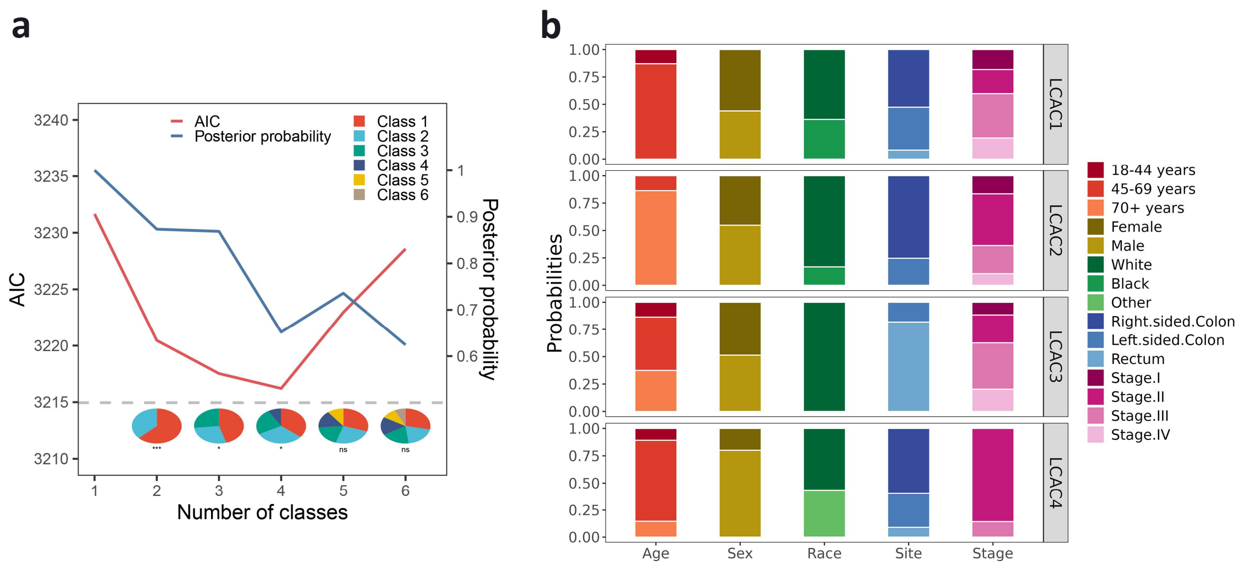

Supplementary Figure 5. LCA-derived classification based on the TCGA database. (a) Model fit assessment for latent class analysis. AIC, Akaike information criteria. (b) Probabilities of indicator variables in each identified class. ***P < 0.001, **P < 0.01, *P < 0.05, ns: not significant.

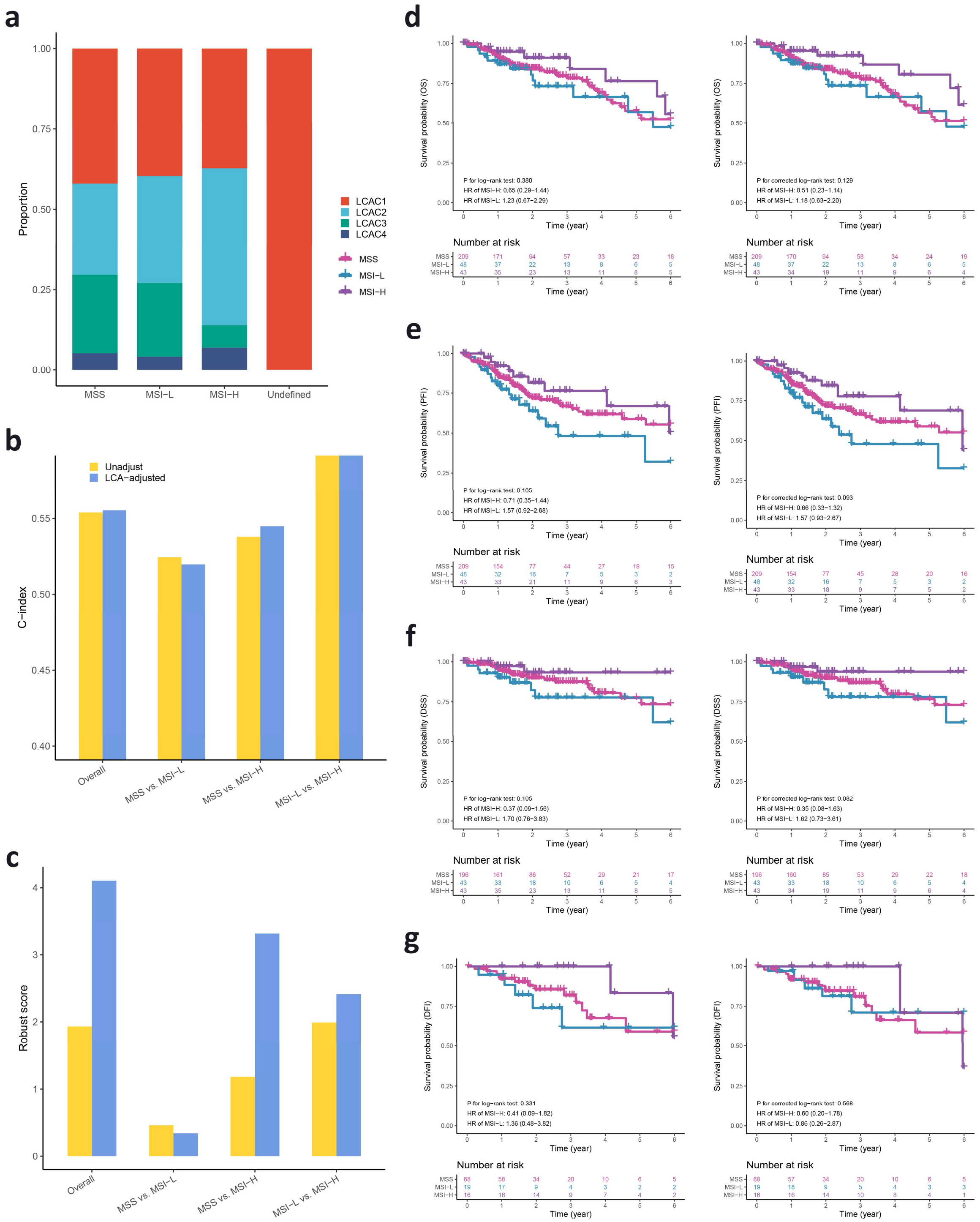

Supplementary Figure 6. Adjustment performance of LCA-derived classification on microsatellite instability (MSI) in the TCGA database. (a) Proportion of LCA-derived classification in each MSI subtype. (b) C-index for models to distinguish survival of MSI subtypes without and with adjustment of LCA-derived classification. (c) Robust score for comparison of survival between MSI subtypes without and with adjustment of LCA-derived classification. Cumulative survival probability (or survival function, survival rate) of CMS subtypes without (left panel) or with (right panel) adjustment of LCA-derived classification, for OS (d), PFI (e), DSS (f), and DFI (g), respectively.

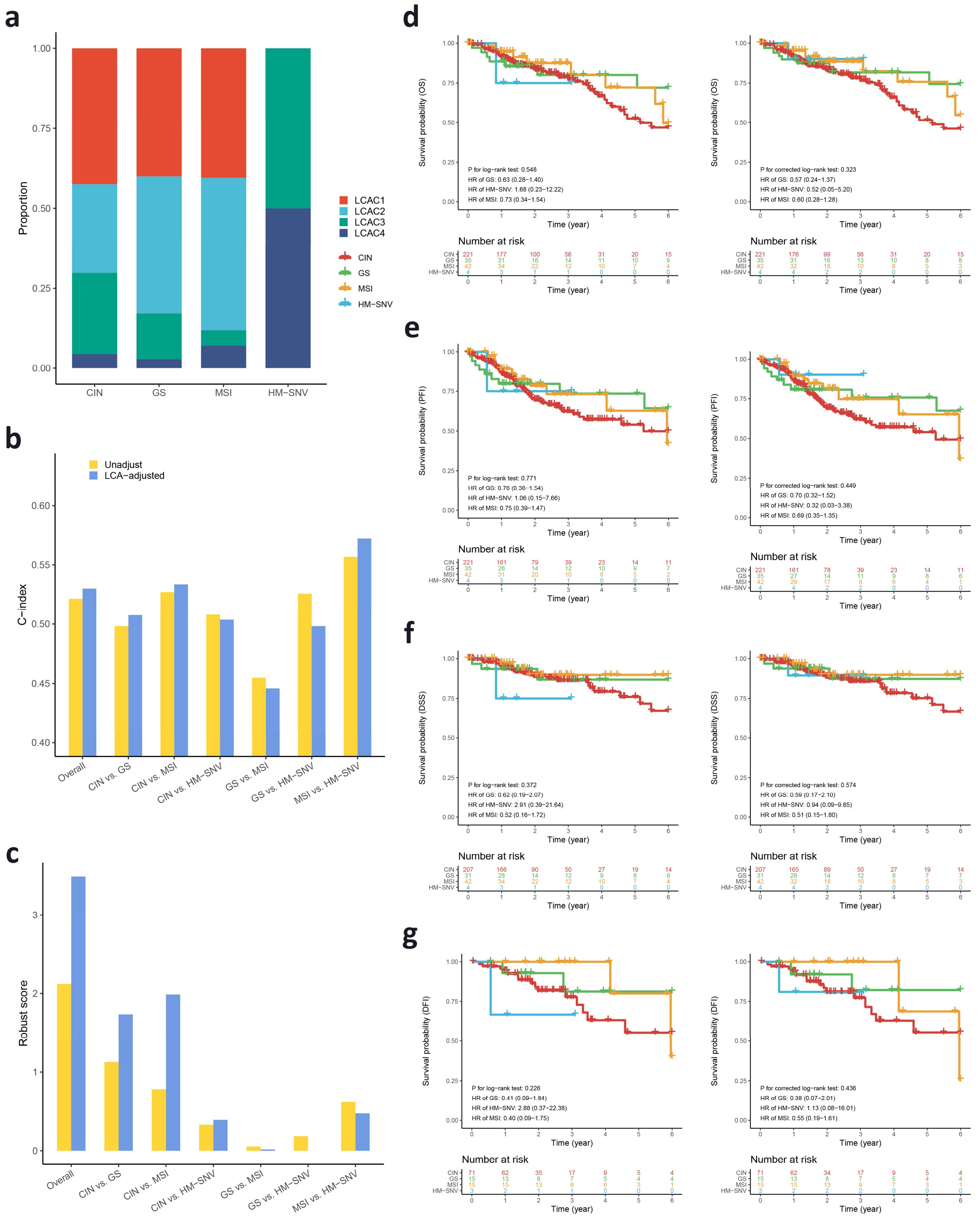

Supplementary Figure 7. Adjustment performance of LCA-derived classification on molecular subtypes in the TCGA database. (a) Proportion of LCA-derived classification in each molecular subtype. (b) C-index for models to distinguish survival of molecular subtypes without and with adjustment of LCA-derived classification. (c) Robust score for comparison of survival between molecular subtypes without and with adjustment of LCA-derived classification. Cumulative survival probability (or survival function, survival rate) of molecular subtypes without (left panel) or with (right panel) adjustment of LCA-derived classification, for OS (d), PFI (e), DSS (f), and DFI (g), respectively.

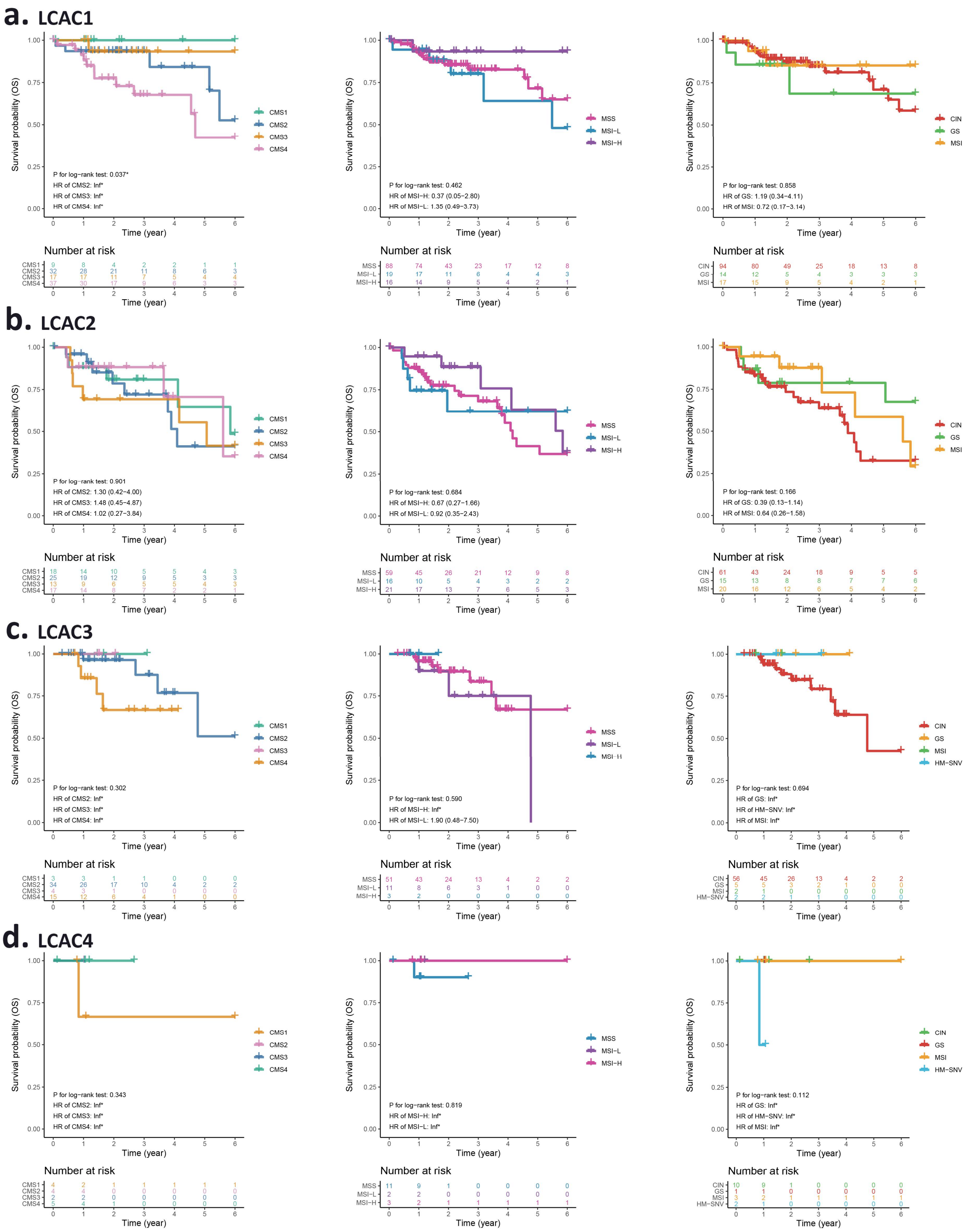

Supplementary Figure 8. Cumulative survival probabilities (OS) of different CRC molecular subtypes in each of the four LCA classes in the TCGA database. Inf*: hazard ratio (HR) not shown because of infinite.

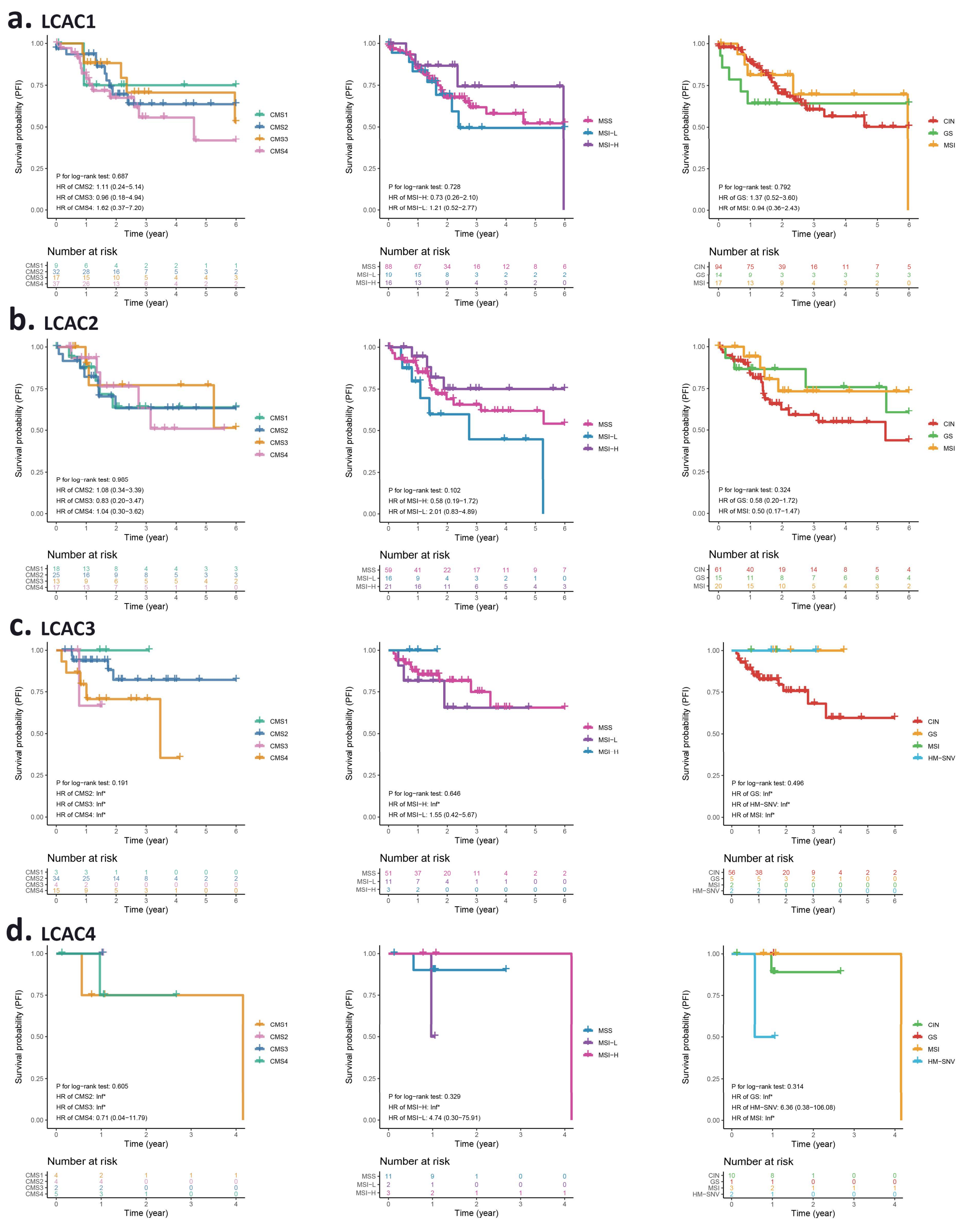

Supplementary Figure 9. Cumulative survival probabilities (PFI) of different CRC molecular subtypes in each of the four LCA classes in the TCGA database. Inf*: hazard ratio (HR) not shown because of infinite.

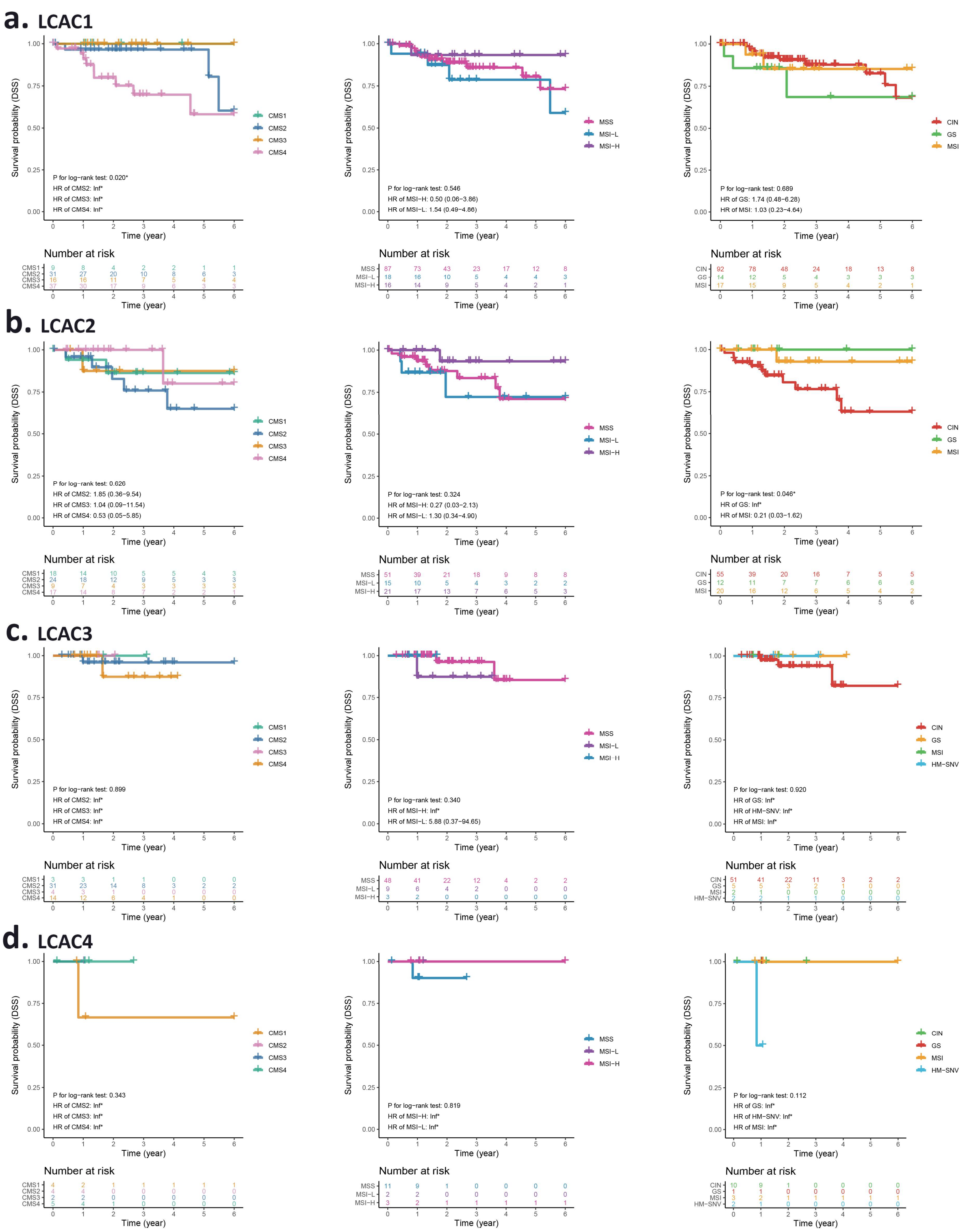

Supplementary Figure 10. Cumulative survival probabilities (DSS) of different CRC molecular subtypes in each of the four LCA classes in the TCGA database. Inf*: hazard ratio (HR) not shown because of infinite.

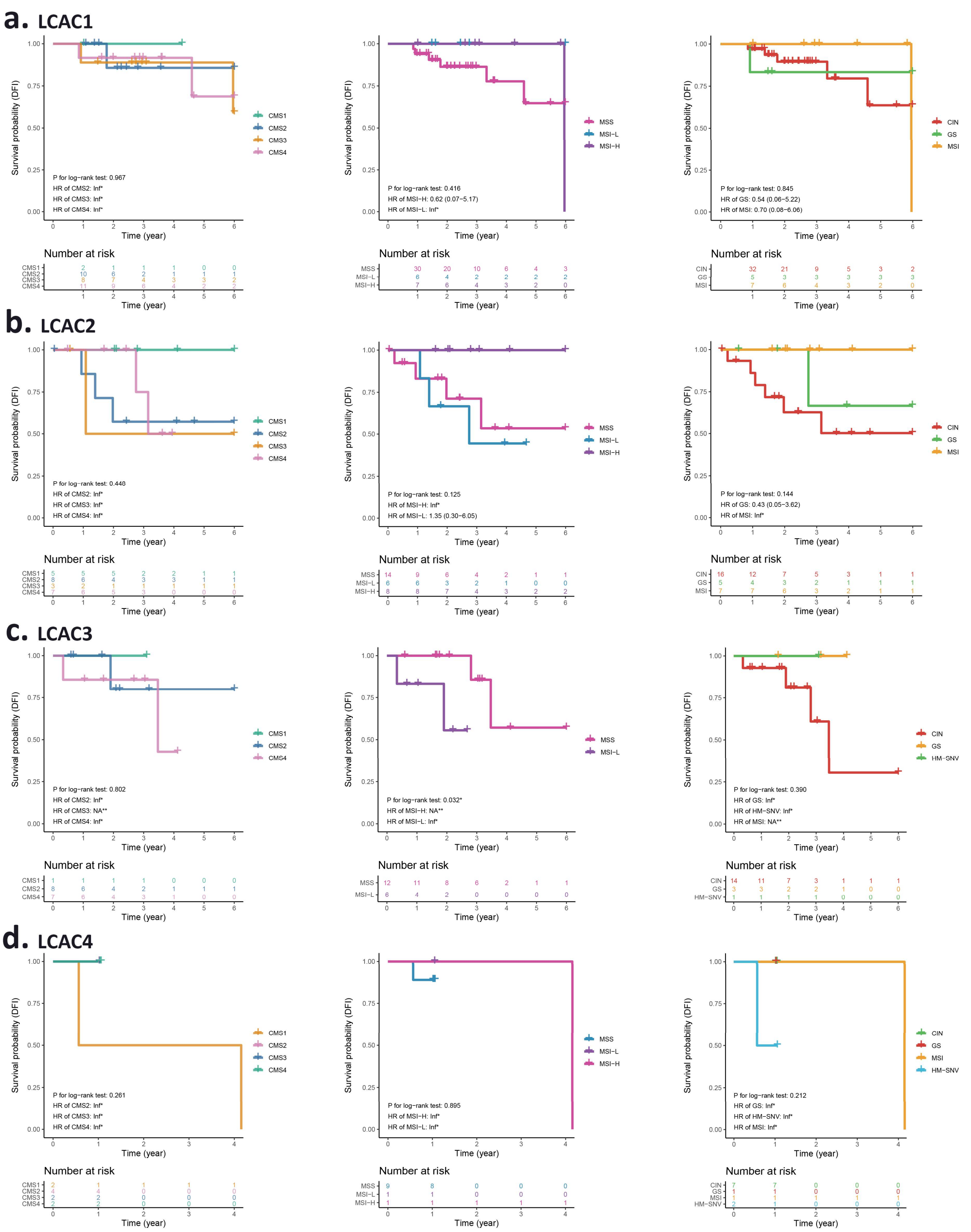

Supplementary Figure 11. Cumulative survival probabilities (DFI) of different CRC molecular subtypes in each of the four LCA classes in the TCGA database. Inf*: hazard ratio (HR) not shown because of infinite. NA**: HR not shown because there were no individuals for this subgroup.

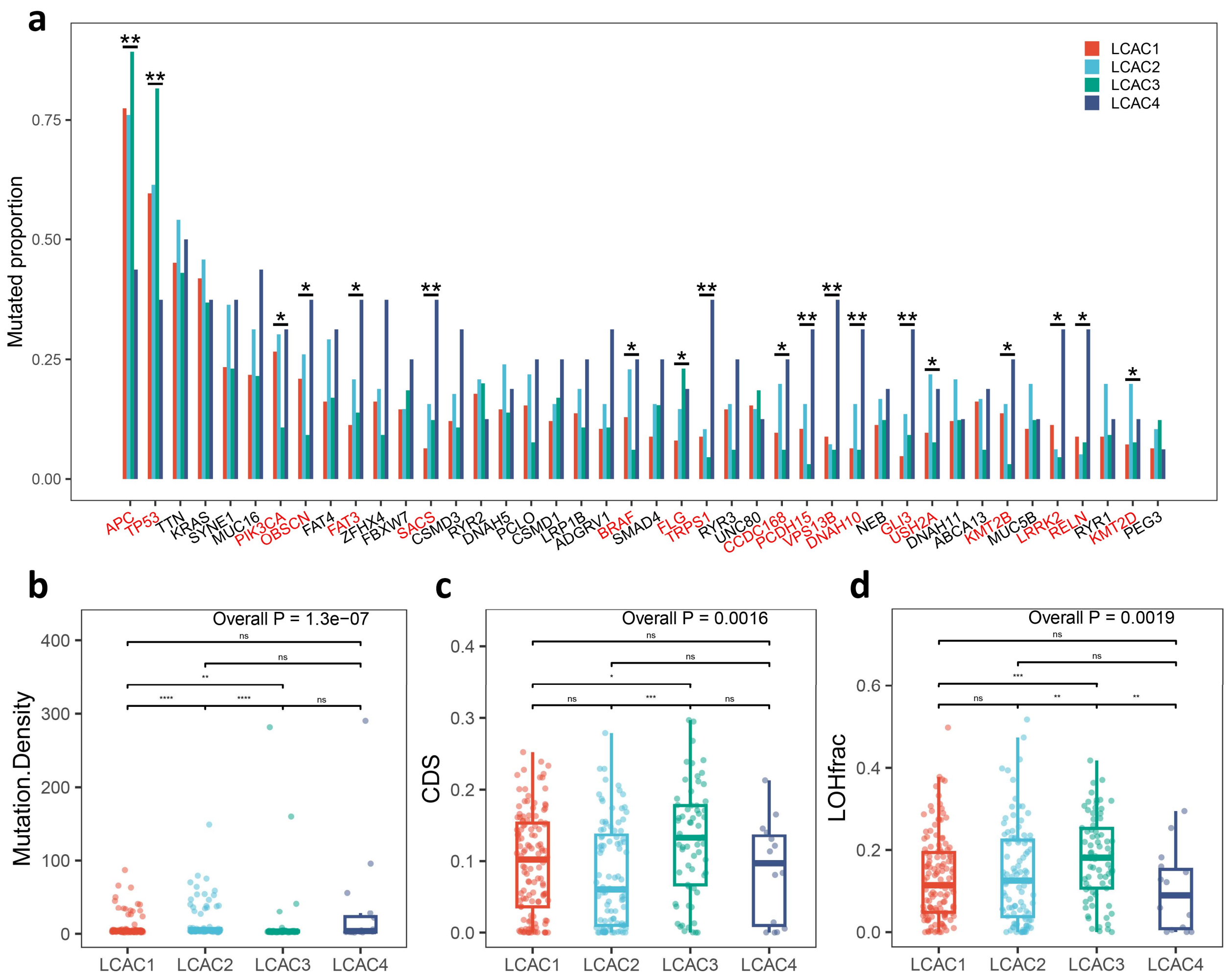

Supplementary Figure 12. Molecular features of LCA-derived classification based on the TCGA dataset. (A) Comparison of mutation frequency among classes. Boxplot of mutation density (B), clonal deletion score (C), and loss of heterozygosity (LOH) fraction (D) in each class. The center line of boxplots denotes the median value (50th percentile), the bounds of box contain the 25th and 75th percentiles of dataset, the upper whisker extends from the upper boundary to the largest value no further than 1.5 * IQR from the upper boundary (where IQR is the inter-quartile range, or distance between the 25th and 75th percentiles), and the lower whisker extends from the lower boundary to the smallest value at most 1.5 * IQR of the lower boundary. ****P < 0.0001, ***P < 0.001, **P < 0.01, *P < 0.05, ns: not significant.
