# Supplementary material for: Latent class analysis-derived classification improves the cancer-specific death stratification of molecular subtyping in colorectal cancer
Source: NPJ Precis Oncol. 2023 Jun 23;7:60. doi: 10.1038/s41698-023-00412-w (PMC10290127; doi:10.1038/s41698-023-00412-w)
Supplement: Supplementary file 2 — REPORTING SUMMARY [file 41698_2023_412_MOESM2_ESM.pdf]

nature portfolio

Corresponding author(s): Qi Zhao

Last updated by author(s): Jun 2, 2023

Reporting Summary

Nature Portfolio wishes to improve the reproducibility of the work that we publish. This form provides structure for consistency and transparency in reporting. For further information on Nature Portfolio policies, see our Editorial Policies and the Editorial Policy Checklist.

Statistics

For all statistical analyses, confirm that the following items are present in the figure legend, table legend, main text, or Methods section.

n/a	Confirmed	
☐	☒	The exact sample size (*n*) for each experimental group/condition, given as a discrete number and unit of measurement
☐	☒	A statement on whether measurements were taken from distinct samples or whether the same sample was measured repeatedly
☐	☒	The statistical test(s) used AND whether they are one- or two-sided<br>*Only common tests should be described solely by name; describe more complex techniques in the Methods section.*
☐	☒	A description of all covariates tested
☐	☒	A description of any assumptions or corrections, such as tests of normality and adjustment for multiple comparisons
☐	☒	A full description of the statistical parameters including central tendency (e.g. means) or other basic estimates (e.g. regression coefficient) AND variation (e.g. standard deviation) or associated estimates of uncertainty (e.g. confidence intervals)
☐	☒	For null hypothesis testing, the test statistic (e.g. *F*, *t*, *r*) with confidence intervals, effect sizes, degrees of freedom and *P* value noted<br>*Give P values as exact values whenever suitable.*
☒	☐	For Bayesian analysis, information on the choice of priors and Markov chain Monte Carlo settings
☒	☐	For hierarchical and complex designs, identification of the appropriate level for tests and full reporting of outcomes
☐	☒	Estimates of effect sizes (e.g. Cohen's *d*, Pearson's *r*), indicating how they were calculated

Our web collection on statistics for biologists contains articles on many of the points above.

Software and code

Policy information about availability of computer code

Data collection	Not applicable. The study involves analysis of existing data. The data analyzed in this study were obtained from SEER Program SEER*Stat Database, the Genomic Data Commons (GDC) program, and the UCSC Xena platform.
Data analysis	All statistical analyses were performed in R version 4.0.3. All code for data cleaning and analysis associated with the current submission is available at https://github.com/wenzhou-cn/LCAC.

For manuscripts utilizing custom algorithms or software that are central to the research but not yet described in published literature, software must be made available to editors and reviewers. We strongly encourage code deposition in a community repository (e.g. GitHub). See the Nature Portfolio guidelines for submitting code & software for further information.

Data

Policy information about availability of data

All manuscripts must include a data availability statement. This statement should provide the following information, where applicable:

- Accession codes, unique identifiers, or web links for publicly available datasets
- A description of any restrictions on data availability
- For clinical datasets or third party data, please ensure that the statement adheres to our policy

The data analyzed in this study were obtained from SEER Program SEER*Stat Database (Incidence - SEER Research Data, 17 Registries, Nov 2022 Sub [2000-2020] - Linked To County Attributes - Time Dependent [1990-2021] Income/Rurality, 1969-2021 Counties, National Cancer Institute, DCCPS, Surveillance Research Program,

released April 2023, based on the November 2022 submission), of which detailed information is available on https://seer.cancer.gov/data-software/documentation/seerstat/nov2022/. A SEER*Stat account is needed to access the SEER Research Data (for personal use), with acknowledge of the SEER Research Data Use Agreement, SEER Treatment Data Limitations, and Best Practices Assurance. The demographic and clinicopathological information of the TCGA cohort were obtained from the Genomic Data Commons (GDC) program which provided by a previous study 73 (https://gdc.cancer.gov/about-data/publications/Pan-GI) and the UCSC Xena platform (Cohort names: “GDC TCGA Colon Cancer” and “GDC TCGA Rectal Cancer” at https://xenabrowser.net/datapages/). The survival information was downloaded using the UCSC Xena platform (Cohort name: “TCGA Colon and Rectal Cancer” at https://xenabrowser.net/datapages/). The somatic mutation calling of the TCGA cohort was downloaded using the GDC Data Transfer Tool (UUID for colon cancer: 70cb1255-ec99-4c08-b482-415f8375be3f, 03652df4-6090-4f5a-a2ff-ee28a37f9301, 70835251-ddd5-4c0d-968e-1791bf6379f6, and 8177ce4f-02d8-4d75-a0d6-1c5450ee08b0; UUID for rectal cancer: ec8ec3ad-f08d-46eb-9571-42806e304b37, faa5f62a-2731-4867-a264-0e85b7074e87, e48ffb82-9208-4be3-8a47-0a1168a07054, and b2689e8f-3b64-4214-8a87-dc7e7cf6fe5e).

Research involving human participants, their data, or biological material

Policy information about studies with human participants or human data. See also policy information about sex, gender (identity/presentation), and sexual orientation and race, ethnicity and racism.

Reporting on sex and gender	Sex information for this study was obtained directly from the existing database, the SEER and the TCGA. The observed indicators used in LCA included patient demographic characteristics and clinicopathological information such as age at diagnosis, sex, race, tumor site, and stage. Sex was assessed as a dichotomous variable (male and female).
Reporting on race, ethnicity, or other socially relevant groupings	Race information for this study was obtained directly from the existing database, the SEER and the TCGA. The observed indicators used in LCA included patient demographic characteristics and clinicopathological information such as age at diagnosis, sex, race, tumor site, and stage. Race was classified as non-Hispanic White, non-Hispanic Black, Hispanic (All Races), and other.
Population characteristics	A total of 491,107 first primary CRC patients were included (Supplementary Table 1, Supplementary Figure 1). Throughout the entire follow-up period, a total of 268,034 patients died, with 43.72% of them attributed to non-CRC causes, especially cardiovascular disease. The range of follow-up period was 0-20.9 years, with a median follow-up of 3.9 years. Characteristics of most of the patients included were male (52.24%), age at diagnosis between 45-69 years (51.84%), non-Hispanic White (68.51%), married (56.71%), residing in metropolitan areas with a population greater than one million (57.16%), income between $50,000-$74,999 (48.19%), right-sided colon tumor (41.17%), stage III (28.40%), grade G2 (70.74%), and adenocarcinoma (72.53%).
Recruitment	The study involves analysis of existing data. For the SEER database, subjects were excluded if their diagnoses were not confirmed by positive histology. We also excluded subjects diagnosed before 18 years old, with staged carcinoma in situ, with tumors located in the appendix, or those with unknown age at diagnosis, race/ethnicity, stage, tumor sites, cause of death, and/or date of death. For the TCGA database, colorectal cancer patients without missing data in age at diagnosis, sex, race/ethnicity, tumor site, and stage were included in this study.
Ethics oversight	Not applicable. The study involves analysis of existing data.

Note that full information on the approval of the study protocol must also be provided in the manuscript.

Field-specific reporting

Please select the one below that is the best fit for your research. If you are not sure, read the appropriate sections before making your selection.

☒ Life sciences ☐ Behavioural & social sciences ☐ Ecological, evolutionary & environmental sciences

For a reference copy of the document with all sections, see nature.com/documents/nr-reporting-summary-flat.pdf

Life sciences study design

All studies must disclose on these points even when the disclosure is negative.

Sample size	A total of 491,107 first primary CRC patients from SEER program were included. A total of 350 CRC patients from TCGA program were included. No statistical method was used to predetermine sample size. Maximal dataset was retained for analysis after data exclusions.
Data exclusions	For the SEER database, subjects were excluded if their diagnoses were not confirmed by positive histology. We also excluded subjects diagnosed before 18 years old, with staged carcinoma in situ, with tumors located in the appendix, or those with unknown age at diagnosis, race/ethnicity, stage, tumor sites, cause of death, and/or date of death. For the TCGA database, colorectal cancer patients without missing data in age at diagnosis, sex, race/ethnicity, tumor site, and stage were included in this study.
Replication	We used the TCGA dataset of 350 CRC patients to verify the consistency of the classification.
Randomization	Not applicable. Randomization was not relevant to this study since it did not involve allocation of experimental groups to a specific intervention.
Blinding	Not applicable. Blinding was not relevant to this study.

Reporting for specific materials, systems and methods

We require information from authors about some types of materials, experimental systems and methods used in many studies. Here, indicate whether each material, system or method listed is relevant to your study. If you are not sure if a list item applies to your research, read the appropriate section before selecting a response.

Materials & experimental systems

n/a	Involved in the study	
☒	☐	Antibodies
☒	☐	Eukaryotic cell lines
☒	☐	Palaeontology and archaeology
☒	☐	Animals and other organisms
☒	☐	Clinical data
☒	☐	Dual use research of concern
☒	☐	Plants

Methods

n/a	Involved in the study	
☒	☐	ChIP-seq
☒	☐	Flow cytometry
☒	☐	MRI-based neuroimaging
